# Conjoint analyses of patients’ preferences for primary care: a systematic review

**DOI:** 10.1186/s12875-022-01822-8

**Published:** 2022-09-09

**Authors:** Audrey Huili Lim, Sock Wen Ng, Xin Rou Teh, Su Miin Ong, Sheamini Sivasampu, Ka Keat Lim

**Affiliations:** 1 Centre for Clinical Outcomes Research, Institute for Clinical Research, National Institutes of Health, Ministry of Health, Shah Alam, Malaysia; 2 School of Life Course & Population Sciences, Faculty of Life Sciences & Medicine, King’s College London, London, UK; 3 National Institute for Health Research (NIHR) Biomedical Research Centre, Guy’s and St Thomas’ NHS Foundation Trust and King’s College London, London, UK

**Keywords:** Discrete choice experiments, Conjoint analysis, Primary care, Patients, Preference, Attributes, Preference heterogeneity, Systematic review

## Abstract

**Background:**

While patients’ preferences in primary care have been examined in numerous conjoint analyses, there has been little systematic effort to synthesise the findings. This review aimed to identify, to organise and to assess the strength of evidence for the attributes and factors associated with preference heterogeneity in conjoint analyses for primary care outpatient visits.

**Methods:**

We searched five bibliographic databases (PubMed, Embase, PsycINFO, Econlit and Scopus) from inception until 15 December 2021, complemented by hand-searching. We included conjoint analyses for primary care outpatient visits. Two reviewers independently screened papers for inclusion and assessed the quality of all included studies using the checklist by ISPOR Task Force for Conjoint Analysis. We categorized the attributes of primary care based on Primary Care Monitoring System framework and factors based on Andersen’s Behavioural Model of Health Services Use. We then assessed the strength of evidence and direction of preference for the attributes of primary care, and factors affecting preference heterogeneity based on study quality and consistency in findings.

**Results:**

Of 35 included studies, most (82.4%) were performed in high-income countries. Each study examined 3–8 attributes, mainly identified through literature reviews (*n* = 25). Only six examined visits for chronic conditions, with the rest on acute or non-specific / other conditions. Process attributes were more commonly examined than structure or outcome attributes. The three most commonly examined attributes were waiting time for appointment, out-of-pocket costs and ability to choose the providers they see. We identified 24/58 attributes with strong or moderate evidence of association with primary care uptake (e.g., various waiting times, out-of-pocket costs) and 4/43 factors with strong evidence of affecting preference heterogeneity (e.g., age, gender).

**Conclusions:**

We found 35 conjoint analyses examining 58 attributes of primary care and 43 factors that potentially affect the preference of these attributes. The attributes and factors, stratified into evidence levels based on study quality and consistency, can guide the design of research or policies to improve patients’ uptake of primary care. We recommend future conjoint analyses to specify the types of visits and to define their attributes clearly, to facilitate consistent understanding among respondents and the design of interventions targeting them.

Word Count: 346/350 words.

**Trial registration:**

On Open Science Framework: https://osf.io/m7ts9

**Supplementary Information:**

The online version contains supplementary material available at 10.1186/s12875-022-01822-8.

## Introduction

Primary care, defined as the first contact a person has with the health system, encompasses a broad range of health services, including preventive, curative and rehabilitative services, that addresses both acute and chronic conditions [1–3]. Internationally, better access to primary care has been associated with better health outcomes and lower total healthcare costs [4]. Thus, not only can primary care meet a broad range of the people’s health needs, it can also provide quality health services to people without resulting in financial hardship [5, 6].

To better address the changing health needs due to ageing population and rising prevalence of chronic conditions, many countries worldwide, including the low and middle-income countries (LMICs) have undertaken initiatives to reform their delivery of primary care [7, 8]. A central idea behind many such reforms is person-centred care that emphasises the value of patients’ views in co-designing and in delivering health care [9, 10]. To co-design and to deliver person-centred care at primary care settings require policy makers and primary care service providers to understand patients’ preferences for health services delivered at primary care.

Conjoint analysis is a stated-preference method that derives the implicit values for an attribute of a product or a service using surveys [11]. In a conjoint analysis survey, respondents are presented hypothetical alternatives of a product or a service characterised (conjointly) by two or more attributes, each over a range of levels, alternatives which they are asked to rank, rate, or choose; a choice-based conjoint analysis where respondents are asked to choose between two or more alternatives is also known as “discrete choice experiment (DCE). Based on how the rankings, ratings or choices differ between the shortlisted attributes or between the alternatives of primary care services characterised by the shortlisted attributes, one could estimate preferences associated with the attributes [11] and use the preferences to predict uptake of the primary care service. Conjoint analyses can also elucidate preference heterogeneity by examining factors (e.g., patient characteristics) that modify the preference (and by extension, the uptake of the primary care service), which would provide insight on how to tailor the service to the characteristics of the target population.

Given its usefulness, numerous conjoint analyses on patients’ preference in primary care have been performed among patients visiting primary care facilities or among public members who are potential users of primary care. The only review of conjoint analyses on patients’ preference in primary care thus far found 18 DCEs (including two on out-of-hour service) performed between 2006 and 2015. The review [12] summarised a list of the attributes examined, organised into three general categories of structure, process and outcome attributes. However, it did not synthesise the direction of preference and the strengths of evidence of the attributes. The review also did not examine factors affecting preference heterogeneity. A synthesis of evidence for primary care attributes and factors affecting preference heterogeneity would advise which attributes or factors should be considered in future research and policy decisions in providing person-centred care at primary care settings.

To address these gaps, our review aims (1) to update the list of primary care attributes and to provide a list of factors affecting preference heterogeneity, focusing on outpatient visits based on all studies since the inception of the databases (2) to categorise the attributes based on a framework developed to describe primary care system [13, 14], and the factors based on a framework of health services utilisation [15], and (3) to synthesise the direction and the strength of evidence of the attributes and the factors affecting preference heterogeneity.

## Methods

This systematic review was prospectively registered on Open Science Framework (https://osf.io/m7ts9) and is reported according to the Preferred Reporting Items for Systematic Reviews and Meta-Analyses (PRISMA) guideline (Appendix 1).

### Search strategy

We conducted systematic searches in five databases (PubMed, Embase, PsycINFO, Econlit and Scopus) from inception until 15 December 2021 using terms related to “primary care” and “preferences”, “conjoint analyses” or “DCE” (Appendix 2); these terms were adapted from the previous review on the same topic [12], as well as other systematic reviews in primary care [16–19] and systematic reviews of discrete choice experiments in healthcare [20–22]. To identify studies that may have been missed from database searches, we also hand-searched Google, included studies from previous review [12], and the reference lists of included studies.

### Inclusion and exclusion criteria

All articles from the database searches were downloaded into EndNote for de-duplication, before being screened for eligibility by two independent reviewers (AHL, SWN) based on titles and abstracts and subsequently, based on full text. Any disagreements were reconciled via consensus and if necessary, involving a third reviewer (XRT or KKL). In cases of no access to full text, we contacted the corresponding authors of the studies and the journals multiple times. If we did not receive any response from the corresponding authors and the journals by the time the manuscript draft was complete, the studies were excluded.

We included studies that used DCEs or conjoint analyses to survey the patients or the general public on preferences for primary care outpatient visits.

We excluded studies that examined preferences on specific treatment (e.g., anti-diabetics), specific services in a clinic (e.g., pharmacy services), services in hospital outpatient clinics or out-of-hour services. Studies on out-of-hours service were excluded because they have evolved in some settings to be delivered over the phone or in tandem with hospital emergency departments, hence cater to patients with perceived urgent problems who are different from the general population who use primary care [23]. The inclusion and exclusion criteria are also summarised in Appendix 3.

### Data extraction

We created a data extraction form and a data dictionary using Microsoft Excel to extract data on study settings (publication year, continent, country’s income level, sources of funding), study design (recruitment setting and methods of survey administration), questionnaire design (the choice contexts, the types of primary care visits, the attributes, methods to identify the attributes and level, the factors affecting preference heterogeneity, methods to generate choice set and whether the study reported design efficiency), study samples (sample size, response rate, age, gender) and analyses (statistical model) from eligible articles. We also extracted the direction of association and statistical significance at *p* < 0.05 for the attributes and the factors affecting preference heterogeneity. Factors affecting preference heterogeneity were identified from study sample characteristics that are associated with latent class memberships (among studies that performed latent class analysis) or characteristics that moderated the associations between attributes and primary care uptake (among studies that performed logit or probit regression analyses). The data extraction form and the data dictionary were pre-tested with two studies by AHL and SWN and feedback was obtained to update the form before use.

### Quality appraisal

The quality of the included studies was appraised using the checklist by ISPOR Task Force for Conjoint Analysis [24]. The checklist is made up of 10 items, each comprising 3 criteria. Each criterion was first evaluated “Yes”, “Partial” or “No” by two independent reviewers (AHL with SMO, or SWN). Based on the extent to which the three criteria were met, each item was then rated “Yes”, “Partial” or “No”. Any disagreements between them were reconciled via consensus, and if necessary, involving a third reviewer (LKK).

### Data analyses

To provide an overview, we tabulated, in numbers and percentages, the study and sample characteristics, including the contexts of the choice questions (hereafter “choice contexts”), the types of primary care visits, the attributes and the factors affecting preference heterogeneity. The choice contexts were categorised based on for whom the primary care services were chosen (self, friend or relative) and if specified, the hypothetical reason the choices were required (e.g., current primary care clinic closes). The types of visits were categorised into visits for major acute, minor acute, chronic, or non-specific / other conditions based on data that emerged from the included studies. “Minor acute” conditions included influenza, urinary tract infections and upper respiratory tract infections while “major acute” conditions included severe lower back pain, “new urgent symptoms”, and perceived severe disease. Meanwhile, “non-specific / other conditions” referred to routine check-ups or conditions that were not explicitly stated and thus unable to be categorised into acute or chronic.

**Fig. 1:**
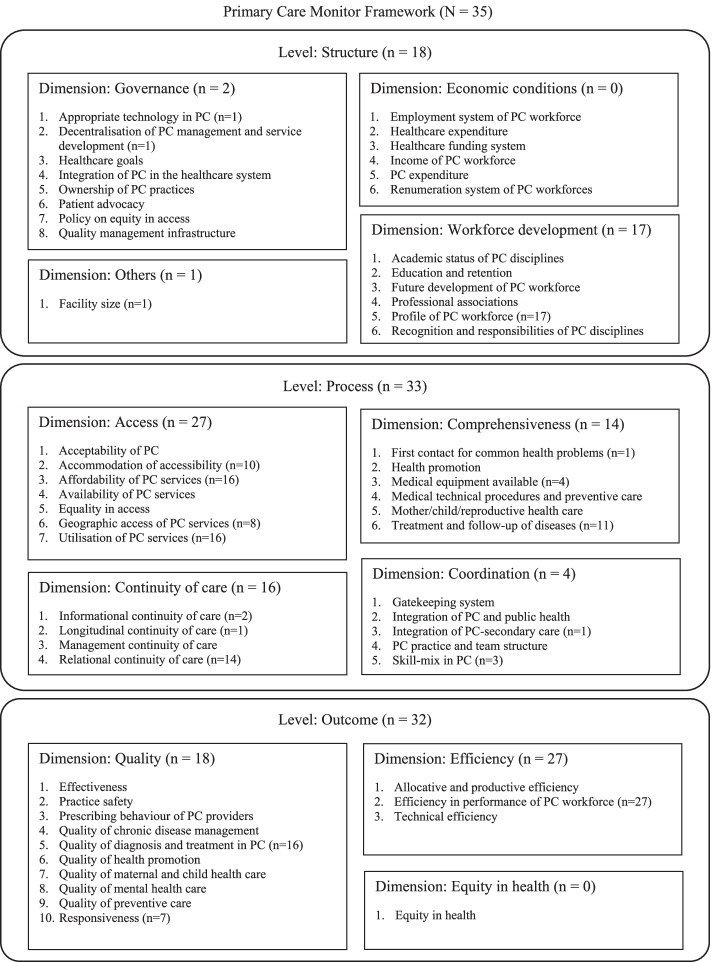
Number of studies examining each level, dimension and feature of the Primary Care (PC) Monitor Framework

Meanwhile, the attributes were categorised into three levels (structure, process, or outcome). Each level was broken down into dimensions and features, based on the Primary Care Monitoring System (PC Monitor) framework. The framework describes primary care systems in three levels of structure, process, and outcome, each further divided into dimensions and features, with a total of 11 dimensions and 57 features. For example, the structure level comprises three dimensions: (a) governance, (b) economic conditions, and (c) workforce development. The governance dimension, for instance, includes the use of appropriate technology, decentralisation, ownership, etc. as its features. Meanwhile, the process level comprises four dimensions: (a) access, (b) continuity of care, (c) coordination of care. and (c) comprehensiveness of care; the outcome level comprises three dimensions: (a) quality of care; (b) efficiency of care; and (c) equity in health [13, 14] (Fig. 1).

Finally, the factors affecting preference heterogeneity were categorised based on Andersen’s Behavioural Model of Health Services Use [15] into predisposing, enabling, health behaviour or need factors.

In the absence of gold standard on what constitutes “high quality”, we considered studies rated either “Yes” or “Partial” across all 10 items as high quality in main analysis and studies rated “Yes” in ≥ 5 out of 10 items as high quality in sensitivity analysis [24].

**Fig. 2:**
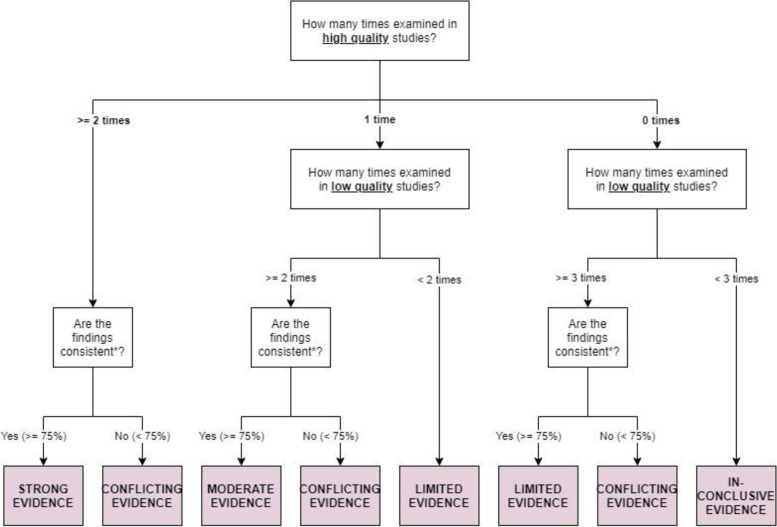
Graphical presentation of the algorithm used to assign evidence level for each attribute and each factor

To synthesise the evidence level, we stratified each attribute and each factor into strong, moderate, limited, conflicting or inconclusive based on study quality and consistency of findings across ≥ 75% studies [25–27]. As illustrated in Fig. 2, an attribute (or a factor) had “strong evidence” if it had been examined ≥ 2 times in studies of high quality, of which ≥ 75% produced consistent findings. If an attribute had been examined once in a high-quality study and ≥ 2 times in low-quality studies with consistent findings, it would be assigned “moderate evidence”. If an attribute had only been examined once in a high- and a low-quality study each or produced consistent findings ≥ 3 times in low-quality studies, it would be assigned “limited evidence”. If an attribute had been examined < 3 times in low-quality studies, the level of evidence would be deemed “inconclusive”. If < 75% of the findings were consistent, the evidence level would be deemed “conflicting” regardless of the study quality. For attributes that were binary (yes/no), ordinal or continuous, consistency accounted for the direction of association (positive, negative, none) as well as statistical significance (at *p* < 0.05) whereas for attributes that were nominal (e.g., choice of providers), consistency accounted for statistical significance; similarly for factors affecting preference heterogeneity. We were unable to account for consistency in the direction for binary (yes/no), ordinal or continuous factors affecting preference heterogeneity due to small number of studies examining the interaction terms of the same factor with the same attribute. This approach of evidence synthesis is commonly used in systematic reviews where meta-analyses are not feasible due to heterogeneity among the included studies. While it has been applied to synthesise evidence levels in systematic reviews of prognostic factors of clinical conditions [25–27], we are not aware of any attempt to apply the approach to synthesise the evidence levels for attributes and / or factors affecting preference heterogeneity in systematic review of conjoint analyses.

All analyses were performed on Microsoft Excel or R version 4.0.5 (The R Foundation for Statistical Computing, Vienna).

## Results

### Study selection

**Fig. 3:**
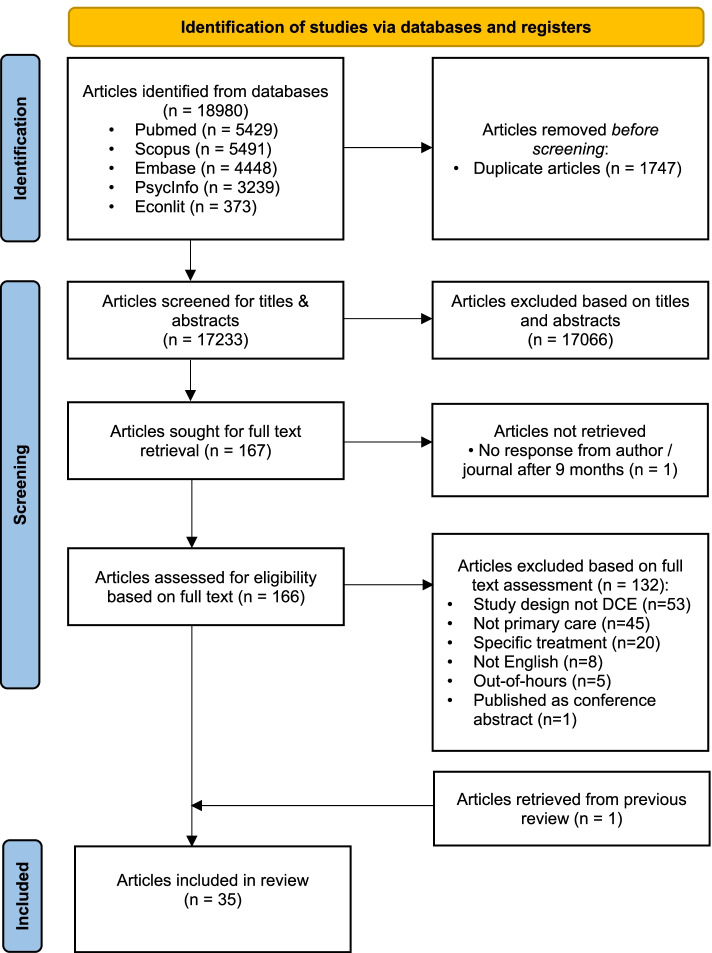
PRISMA flow diagram

The search strategy identified 18,980 articles (Fig. 3), of which 17,233 were unique. After screening their titles and abstracts, 166 were retrieved for full text screening, from which 132 were excluded because they were not DCEs (*n* = 53), were not on primary care (*n* = 45), examined specific treatment (*n* = 20), not English (*n* = 8), examined preferences for out-of-hours treatment (*n* = 5), or conference abstract (*n* = 1). One additional article [28] was retrieved from the previous review [12]. For one abstract that may be eligible based on title and abstract [29], we had to contact the author and the journal via their contact emails and ResearchGate accounts for the full-text but did not receive a reply despite five attempts over a span of nine months. This gave 35 eligible articles for extraction, of which two were rating-based conjoint analyses, and the rest choice-based conjoint analysis or DCEs.

### Study and sample characteristics

Table 1 summarises the study and sample characteristics, with details for each study in Appendix 4. The studies were mostly published after 2010 (60.0%), in Europe (65.7%), from high-income countries (82.9%). Among studies that reported funding sources (71.4%), government funding dominated (45.7%). Study samples were recruited from primary care facilities (54.3%) or the community (42.9%), most of whom self-completed the questionnaires (62.9%). These studies recruited on average 881.8 respondents, with 62.8% response rates. The respondents, with 51.6 years-old mean age, comprised of 41.9% men.

**Table 1:** Characteristics of included studies (*N* = 35)

Characteristics			Characteristics		
***Study settings***	**n**	**%**	***Questionnaire design***	**n**	**%**
**Publication year**			**Choice contexts**^**a**^		
2010–2022	21	60.0	Choosing primary care for self (not specified)	31	88.6
1997–2009	14	40.0	Choosing primary care for self when the current one closes	3	8.6
			Choosing primary care for self after moving to a new city	1	2.9
**Continent**			Choosing primary care for a friend / relative	1	2.9
Europe	23	65.7			
Asia	5	14.3	**Types of visits**^**a**^		
North America	4	11.4	Acute: minor	19	54.3
Australia & New Zealand	2	5.7	Non-specific / other^c^	16	45.7
Africa	1	2.9	Chronic	6	17.1
			Acute: major	4	11.4
**Country’s income level**^**b**^					
High income	29	82.9	**Types of attributes**^**a**^		
Low & middle income	6	17.1	Process	33	94.3
			Outcomes	32	91.4
**Sources of funding**			Structure	18	51.4
Government	16	45.7			
Not reported	10	28.6	**Methods to identify attributes & levels**^**a**^		
Independent organization	5	14.3	Literature review	25	71.4
Academic institution	4	11.4	Qualitative research	22	62.9
			Not reported	4	11.4
**Study samples**^**d**^	**Mean**	**SE**	Policy	3	8.6
Sample size	881.8	739.3	Others	3	8.6
Response rate (%)	62.8	22.9	Expert opinion	2	5.7
Age	51.6	8.7			
Percentage of men (%)	41.9	8.7	**Factors affecting preference heterogeneitya**^e^		
			Did not examine any factor	19	54.3
**Type of conjoint analysis**	**n**	**%**	Predisposing characteristics	10	28.6
Choice-based	33	94.3	Enabling resources	9	25.7
Rating-based	2	5.7	Needs	5	14.3
			Health behaviour	2	5.7
***Study design***	**n**	**%**			
**Recruitment setting**			**Methods to generate choice set**		
Primary care facilities	19	54.3	Software	17	48.6
Community	15	42.9	Not reported	16	45.7
Not reported	1	2.9	Catalogue	2	5.7
**Survey administration**			**Reported design efficiency**^f^		
Self-completed	22	62.9	D-efficient	19	54.3
Interviewer administered	7	20.0	Not reported	16	45.7
Computerized interview	3	8.6			
Computer aided telephone	2	5.7	***Study quality***^**g**^	**n**	**%**
interview			**Main analysis**		
Self-completed & Interviewer administered	1	2.9	High	29	82.8
			Low	6	17.1
***Study analyses***	**n**	**%**	**Sensitivity analysis**		
**Statistical models**^**a**^			High	25	71.4
Logit	26	74.5	Low	10	28.6
Probit	8	22.9			
Latent class analyses	3	8.6			
Others^h^	2	5.7			

^a^* Sums to* > *100% as a study may report / examine more than one of these characteristics*

^b^* Categorised based on The World Bank classification on 21 May 2021 at (*https://datahelpdesk.worldbank.org/knowledgebase/articles/906519-world-bank-country-and-lending-groups*)*

^c^* Six studies specified other reasons for visits e.g., general consultation, annual check-up, and appointments for other family members. The remaining nine studies did not specify the reason for visits*

^d^* Not all studies reported all study characteristics: all 35/35 studies reported sample size, 24/35 reported response rate, 21/35 reported mean age of respondents, 31/35 studies reported percentage of men*

^e^* The factors were based on the Anderson model of healthcare utilization, which categorizes factors affecting healthcare utilization into predisposing characteristics (e.g., age), enabling resources (e.g., income level), needs (e.g., health status) and health behavior (e.g., utilization of healthcare)*

^f^* D-efficiency score indicates the extent to which the studies are balanced and orthogonal. Perfectly efficient designs are balanced (each level appears equally often within an attribute) and orthogonal (each pair of levels appears equally often across all pairs of attributes within the design)*

^g^* Based on the Conjoint Analysis Applications in Health – a Checklist: A Report of the ISPOR Good Research Practices for Conjoint Analysis Task Force. In the main analysis, studies that fulfilled all the items in the checklist (either partially or completely) were considered of acceptable quality. In sensitivity analysis, only studies that completely fulfilled at least 5 items out of 10 in the checklist were considered of acceptable quality*

^h^* Other statistical models are Hierarchical Bayes estimation and fractional replication methodology in the Categories module of SPSS*

The studies examined minor acute (54.3%), non-specific / other (45.7%), chronic (17.1%) and / or major acute (11.4%) conditions. They more frequently used process (94.3%) or outcome (91.4) than structure attributes (51.4%), predominantly identified through literature review (71.4%). Among the 16 studies that investigated factors affecting preference heterogeneity, they most investigated predisposing characteristics (28.6%), followed by enabling resources (25.7%), needs (14.3%) and health behaviour (5.7%). As for statistical analysis, logit model (74.5%) was the most widely used.

### Quality appraisal

Study quality was determined based on the number of items rated “Yes” for each study. Including one study that received only “Yes” ratings, 29/35 studies had “Yes” or “Partial” across all 10 items; these studies were considered high quality in main analysis. Meanwhile, 25/35 studies received ≥ 5 “Yes” ratings and were considered high quality in sensitivity analysis.

Only 4/10 items received at least one “No” – “choice of attributes and levels supported by evidence” (3/35 studies were rated “No”), “choice of experimental design justified and evaluated” (2/35 “No”), “appropriate statistical analyses and model estimations” (2/35 “No”) and “appropriate design of data collection instrument” (1/35 “No”) (Appendix 5).

### Attributes of primary care

Overall, the 35 included studies examined 58 unique primary care attributes 183 times (average 5.2 attributes per study). These attributes fell into 3 levels, 9 dimensions and 19 features of primary care of the PC Monitor framework (Fig. 1, Appendix 6).

Among the 3 levels of primary care, process had the largest number of unique attributes (34) across 4 dimensions (access, comprehensiveness, continuity, and coordination) and 12 features; outcome had 19 unique attributes across 2 dimensions (quality, efficiency) and 3 features; structure had 5 unique attributes across 3 dimensions (governance, workforce, others) and 4 features. Relational continuity of care was the most examined feature within the process level, efficiency in the performance of primary care workforce was the most examined feature within the outcome level, whereas profile of workforce was the most examined feature within the structure level (Fig. 1).

Across all levels, dimensions, and features of primary care, the ten most frequently examined attributes were waiting time for appointment (20 studies), out-of-pocket cost (15 studies), ability to choose the providers they see (15 studies), length of consultation time (12 studies), waiting time at clinic (10 studies) involvement in decision making (10 studies), amount of information received during consultation (8 studies), quality of the physical exam (7 studies), depth of the explanation (6 studies), and convenience of appointment time (5 studies) (Appendix 7).

Based on all 35 included studies regardless of type of visits, of the 58 attributes, none had inconclusive or conflicting evidence, but 21 had strong, 3 had moderate and 34 had limited strength of evidence (Table 2a). Most of the attributes, listed in Table 3, either positively or negatively influenced preference for primary care. For example, higher experience of care providers, availability of a convenient appointment time, better communication skills, better drug availability, longer consultation time, extended opening hours, amount of information received are associated with higher preference of primary care, whereas longer distance, higher out-of-pocket cost and longer waiting time are associated with lower preference; these attributes have strong or moderate strength of evidence in the main analyses and retained their strengths of evidence in the sensitivity analyses, except for drug availability for which the strength of evidence became limited. On the other hand, some attributes in the main analyses have limited strength of evidence of positively influencing preference (e.g., clinic managed by the government, availability of home visits, opening at lunch time or more days in a week, multidisciplinary care) or negatively influencing preference (e.g., clinics seeking voluntary contribution in addition to out-of-pocket cost, waiting time for referral). Finally, a minority of attributes, for instance, amount of billing problems, facility size, and provision of preventive care by the facility were found to have no association with a preference of primary care, although their evidence are also of limited strength.

**Table 2:** (a) Number of attributes and (b) number of factors affecting preference heterogeneity

Evidence Level	Number of Attributes / Factors Affecting Preference Heterogeneity Overall or by Type of Visits				
	**Overall**	**Acute: Minor Conditions**	**Acute: Major Conditions**	**Chronic Conditions**	**Non-specific / Other Conditions**^**a**^
**(a) Attributes of primary care**					
**Main Analyses**					
** Strong**	21	15	3	6	10
** Moderate**	3	-	-	-	3
** Limited**	34	21	12	14	22
** Conflicting**	-	-	-	-	-
** Inconclusive**	-	2	5	-	4
** Total**	58	38	20	20	39
**Sensitivity Analyses**					
** Strong**	20	12	3	6	9
** Moderate**	3	1	-	-	4
** Limited**	29	20	12	14	19
** Conflicting**	-	-	-	-	-
** Inconclusive**	6	5	5	-	7
** Total**	58	38	20	20	39
**(b) Factors affecting preference heterogeneity**					
**Main Analyses**					
** Strong**	4	2	-	-	2
** Moderate**	-	-	-	-	-
** Limited**	31	23	4	7	7
** Conflicting**	3	5	-	-	1
** Inconclusive**	5	5	-	-	-
** Total**	43	35	4	7	10
**Sensitivity Analyses**					
** Strong**	4	3	-	-	-
** Moderate**	-	-	-	-	-
** Limited**	28	23	4	7	7
** Conflicting**	3	4	-	-	-
** Inconclusive**	8	5	-	-	3
** Total**	43	35	4	7	10

^a^This includes six studies that specified other reasons for visits e.g., general consultation, annual check-up, and appointments for other family members. The remaining nine studies did not specify the reason for visits

**Table 3:** The 58 attributes examined in the included studies, according to the overall strength of evidence and the levels of primary care in main analyses

Strength of Evidence	Attributes according to levels of primary care^a^		
	Structure	Process	Outcome
Strong	1.Ability to choose the providers they see (NA)	1.Availability of convenient appointment time ( +)	1.Amount of information received during consultation ( +)
	2.Experience of care provider ( +)	2.Communication skills of healthcare provider ( +)	2.Depth of explanation ( +)
		3.Courtesy and respect for the patient ( +)	3.Consideration of patient’s perspective ( +)
		4.Distance to practice – time (-)	4.Involvement in decision making ( +)
		5.Drug availability ( +)	5.Likelihood of having illness cured ( +)
		6.Length of consultation time ( +)	6.Waiting time – appointment (-)
		7.Opening hours – extended ( +)	7.Waiting time – clinic (-)
		8.Out-of-pocket cost (-)	
		9.Quality of the physical exam ( +)	
		10.See provider you know ( +)	
		11.Treatment measures (NA)	
		12.Type of consultation (NA)	
Moderate	None	1.Distance to practice – miles/km (-)	1.Waiting time – telephone (-)
		2.Opening hours – weekend ( +)	
Limited	1.Amount of billing problems (0)	1.Availability of home visits ( +)	1.Attention to personal situation ( +)
	2.Facility size (0)	2.Care for ongoing health conditions (chronic care) (0)	2.Provider’s interpersonal manner ( +)
	3.Management of clinic by government ( +)	3.Familiarity with healthcare personnel ( +)	3.Trustworthiness of the provider ( +)
		4.Friendliness and helpfulness of staff ( +)	4.Reassurance from the provider (+)
		5.General condition of medical equipment ( +)	5.Support for emotional distress ( +)
		6.Insurance reimbursement ( +)	6.Provider notices what you say about your health (legitimation) ( +)
		7.Limited provision of acute care (0)	7.Entire time spent to seek and obtain treatment (0)
		8.Availability of modern diagnostic equipment ( +)	8.Patient satisfaction ( +)
		9.Multidisciplinary care ( +)	9.Waiting time – general (-)
		10.Opening hours – lunchtime ( +)	10.Waiting time – referral (-)
		11.Opening hours – number of days ( +)	11.Whether practice meets your specific health needs ( +)
		12.Personal connection in the facility (0)	
		13.Provider’s knowledge of the patient ( +)	
		14.Practice knows your local services (-)	
		15.Primary care work model ( +)	
		16.Prior expert treatment ( +)	
		17.Provision of preventive care (0)	
		18.See person who has information about your medical history ( +)	
		19.Voluntary contribution (in addition to out-of-pocket cost) (-)	
		20.Availability of technical equipment ( +)	

^a^*Only continuous or ordinal attributes have direction assigned:“0” indicates no association, “* + *” indicates positive association, “-” indicates negative association e.g. increased drug availability is preferred whereas increased waiting time is not. “NA” indicates not applicable*

The number of attributes with strong or moderate evidence decreased when the evidence was stratified by the type of visits, with some attributes becoming inconclusive (Table 2a). The full list of attributes is available in Appendix 7, including how their strengths of evidence varied with the type of visits.

### Factors affecting preference heterogeneity of primary care

The 16 studies examined 43 unique factors affecting preference heterogeneity (Table 2b) 196 times (average 12.3 factors per study) – enabling resources (22 factors), needs factors (12 factors), predisposing characteristics (7 factors), and health behaviour (2 factors). Of these, only 4 had strong evidence of affecting preference heterogeneity of primary care (Table 4), i.e., age, gender, employment status, and income; all retained their strength of evidence in sensitivity analysis. Older respondents preferred lower out-of-pocket cost [30, 31] and to choose their own healthcare provider [32–34] while younger respondents preferred shorter waiting times [31, 35]. Meanwhile, female respondents preferred to choose their own healthcare provider [33, 34, 36] and better quality physical examination [31]. Patients who are employed were more willing to pay higher out-of-pocket cost [30] but preferred shorter waiting times [34], likewise for those with higher incomes [37]. The remaining factors had limited (*n* = 31), inconclusive (*n* = 5) or conflicting (*n* = 3) evidence of affecting preference heterogeneity of primary care. The full list of factors is available in Appendix 8, including how their strengths of evidence varied with the type of visits.

**Table 4:** The 43 factors affecting preference heterogeneity examined in the included studies, according to their overall strength of evidence and Andersen’s framework in main analyses

Strength of Evidence	Factors affecting preference heterogeneity, according to Andersen’s framework			
	**Enabling**	**Health Behaviors**	**Need**	**Predisposing**
Strong	1.Employment status	None	None	1.Age
	2.Income level			2.Gender
Limited	1.Activity if not visiting doctor: Attending college	1.Facility visiting experience: Higher levels	1.Appointment for a child	1.Marital status
	2.Activity if not visiting doctor: Cleaning house		2.Appointment for another person	2.Number of family members
	3.Activity if not visiting doctor: Looking after children		3.Frequency of GP Visits in the last year: < 3 times	3.Region
	4.Activity if not visiting doctor: Other activity		4.Reason for appointment: Emergency	4.You trust in your GP: Yes
	5.Activity if not visiting doctor: Work		5.Reason for appointment: Long standing physical problem	
	6.Advice was given by GP in current visit: Yes		6.Reason for appointment: New problem	
	7.Car ownership: Yes		7.Reason for appointment: Psychological problem	
	8.Carer status: Yes		8.Severity of symptoms	
	9.Current GP works with another GP			
	10.Ever had second opinion			
	11.GP involved you in the decision: Yes			
	12.GP listened to you carefully: Yes			
	13.Insurance type: High premium			
	14.Living alone: Yes			
	15.Prior experience putting off seeking care from GP: Yes			
Inconclusive	1.Current waiting time at present appointment	1.Time since last visit	1.Technical equipment available	None
	2.Distance to health care centre		2.Reason to see GP in current visit: general / minor illness	
	3.Present registration with GP			
	4.Decision making at last visit			
	5.GP provided a lot of information at last visit			
Conflicting	None	None	1.Chronic disease status: Yes	1.Education level
			2.Health Status: Poor	

## Discussion

### Summary

To provide person-centred care, primary care provision should align with patients’ preferences. The preferences of patients as well as public members who could be patients have been examined in numerous conjoint analyses. However, no systematic effort has been undertaken to synthesise their findings. To address this gap, our systematic review identified, organised, and assessed the evidence level of the attributes examined for patients’ preferences in primary care as well as the factors affecting these preferences. The 35 included conjoint analyses had similar characteristics – most were published in the last decade (since 2010), by high-income countries in Europe based on samples recruited from primary care facilities seeking to elicit preferences on visits for acute or non-specific / other conditions. Thus, it may not be surprising that despite spanning diverse levels, dimensions, and features of primary care, none of the 58 attributes was found to have conflicting evidence. Instead, 24 had strong or moderate evidence of an association with preference for primary care, while the remaining 34 attributes had limited evidence of an association or no association. Similarly for the factors affecting preference heterogeneity, albeit with smaller number of studies and only 4 factors found to have strong or moderate evidence.

Process of care, which had the highest number of unique attributes (vs structure and outcomes), was the most studied level of primary care. As no single unique attribute dominated the list, this indicates more varied priorities in selecting process attributes. Conversely, the lack of interest on structure of care (the lowest number of unique attributes) may be due to structural attributes being less observable by the public and less amenable by the policy makers in the short-term.

Meanwhile, the absence of attributes with conflicting evidence from our syntheses implies that patients or public members generally have consistent preference, at least within the contexts examined by the included studies. The consistency suggests the feasibility to improve primary care uptake by changing the attributes in the direction associated with a higher preference. Based on our review, examples of such attributes may be the providers’ communication skills (strong evidence for all visits except that for chronic conditions), quality of the physical examinations (strong evidence for minor acute conditions) and opening hours in the weekend (strong evidence for other / non-specific visits). On the other hand, our review also found some studies reporting attributes with subjective or unclear definition e.g., “best care” in one of the included studies [38]. Such attributes are likely challenging to operationalise and to target in policy interventions, as they may be understood differently by different respondents. To facilitate consistent understanding and the design of policy interventions, [39, 40], we recommend future studies to clearly define and present their attributes (e.g. as a table in Wang et al. [41]).

As few studies examined factors affecting preference heterogeneity, most factors had either limited or inconclusive evidence. Out of the 43 unique factors, only four were examined across enough studies to have strong evidence affecting preference heterogeneity (age, gender, employment status, and income). Younger respondents and those with higher incomes may have lower preference for long waiting times for acute conditions [35] due to perceived lower value of a visit [42], while older respondents prefer lower out-of-pocket costs [30, 37] possibly due to growing financial constraints [43] or healthcare expenditure with age [44]. Meanwhile, women respondents may prefer to choose their own providers [33], as they are likely to trust female physicians more [45] and are more comfortable with female physicians [46, 47]. On the other hand, three factors were found to have conflicting evidence (education level, health status, and chronic disease status), which may be due to the same factor interacting differently with different attributes. For instance, those with chronic diseases were found to prefer more information on their condition but also less involvement in their treatment [48]. Hence, unlike that for attributes, we could not examine the direction of association for the factors affecting preference heterogeneity, which should be explored further in future conjoint analyses.

### Comparison with existing literature

The only other review [12] on patients’ preferences in primary care encompassed three databases between 2006 and 2015, compared to five databases without date restriction (until 15 December 2021) in our review. This gives us more eligible studies (35 vs 18) and unique attributes (58 vs 30). Of the 18 studies from the previous review [12], 16 were included in our current review (15 of which appeared on our database searches); the remaining two [49, 50] were excluded as they examined out-of-hour service. In terms of findings, the earlier review [12] found structure attributes to be the most common whereas our review found process attributes to be predominant. This difference in findings is due to both reviews using different approaches to definitions in categorising the attributes, the earlier review [12] followed the definitions in Donabedian’s model for quality of health care [51] whereas we followed that in the PC Monitor framework [13, 14] which was specifically designed for primary care and allowed us to sub-categorise each attribute into dimensions and features. This resulted in some attributes e.g., opening hours, cost and distance that were “structure” in the earlier review [12] but were considered “process” in our review.

In addition to a list of attributes, our review also generates additional insights by (1) examining the factors affecting heterogeneity, (2) appraising the quality of included studies and (3) synthesising, based on study quality and consistency in findings, the evidence levels of the attributes and the factors affecting preference heterogeneity overall, and by the types of visits. Our findings on the attributes, their evidence level and direction of association largely corroborate findings from other quantitative or qualitative studies on barriers and facilitators on access to primary care that found higher preference for shorter travel distance to health facility [52], shorter waiting time [53, 54], lower out-of-pocket costs [55], being treated with respect and having their own choice of healthcare provider [56]. Similarly for our findings on the factors affecting preference heterogeneity where female respondents preferred to choose their healthcare provider who they were more comfortable with [46, 47], while older respondents preferred to choose healthcare provider but placed higher emphasis on the doctor making decisions [57]. Those with higher incomes were also willing to pay more for treatment than respondents with lower incomes [57].

### Strengths and limitations

Our findings should be interpreted alongside several limitations. First, the categories of attributes are based on the PC Monitor framework, which may have different definitions than other frameworks for primary care services [13]. However, as the framework was developed based on systematic review [13, 14], it increases the generalisability of our findings to other settings. Second, some attributes may fit under > 1 category. For instance, “quality of the physical exam” reported in Cheraghi-Sohi et al. [58] and Kruk et al. [31] was categorised in “treatment and follow-up of diagnosis” feature of primary care (Appendix 6), although it may also fit into “quality of diagnosis and treatment in primary care”. However, we categorised each attribute only to one level, one domain and one feature, for ease of interpretation. Next, as we synthesised evidence only from published literature, our findings on the evidence levels may be susceptible to publication bias. In addition, as we extracted findings only from the final model, our findings on the evidence levels may also be sensitive to model selection by the respective studies. Besides that, the small number of studies that examined factors affecting preference heterogeneity only allowed us to synthesise the overall evidence levels of these factors, rather than based on how they interact with different attributes, which can be explored in future conjoint analyses or future reviews. Finally, we only included conjoint analyses examining primary care outpatient visits. Hence, our findings may not generalise to other services that may be considered primary care e.g., antenatal care [59, 60] or pharmacy services [61].

Despite the limitations, the syntheses of evidence levels for the attributes and the factors affecting preference heterogeneity are our main strengths. To our knowledge, this has only been done on systematic reviews of prognostic factors [25–27] but not by any systematic review of DCEs.

### Implications for research and/or practice

For research, our findings may advise the choice of attributes and factors affecting preference heterogeneity in future conjoint analyses. For instance, future conjoint analyses may focus on attributes with limited or inconclusive evidence, or attributes in levels, dimensions or features of primary care that have been less studied. We also found a paucity of evidence for chronic conditions or in LMICs apart from China, despite the importance of primary care in meeting the preventive and curative care needs of patients in chronic conditions including in LMICs. In addressing these gaps, we recommend future conjoint analyses to specify the types of visits, as our findings suggest patients’ preferences may differ for different types of primary care visits.

For policy, our findings provide an evidence-based list of attributes to design primary care services for optimal uptake, at the local, regional, and national levels. At the local level, the attributes with strong or moderate evidence suggest that extending opening hours as well as allowing patients to choose their own providers or see a provider they are familiar with would improve the uptake of primary care services. Similarly, proactive management of the waiting time to get an appointment or waiting time at the clinic may also help. Healthcare providers may also be provided with trainings on communication skill, including how to get patients involved in their treatment decisions. At the regional or the national level, new primary care facilities should ideally be built in a location within reasonable distance travel time from nearby community, with services available at reasonable out-of-pocket cost. It will be up to the policy makers to determine which attributes should be prioritised first based on local context, whether as part of an ongoing changes or part of a larger reform.

## Conclusion

Our review found 35 studies that examined 58 attributes and 43 factors that potentially affect patients’ preference in primary care, which we categorised based on PC Monitor framework and synthesised the strength of evidence based on study quality and consistency of study findings across studies. The lists of attributes and factors with their evidence levels can guide policies to improve patients’ uptake of primary care and future DCE studies in this area. Due to the lack of conjoint analyses performed in LMICs or examining visits for chronic conditions, we recommend future DCEs to look into these. In addressing any research gaps on preference for primary care outpatient visits, they should specify the types of visits and define their attributes clearly, to facilitate the design of interventions to target these attributes.

## Supplementary Information

**Additional file 1:** **Appendix 1.** PRISMA checklist. **Appendix 2.** Search strategies. **Appendix 3.** List of inclusion and exclusion criteria. **Appendix 4.** Detailed characteristics of included studies (include quality rating for each paper). **Appendix 5.** Methodological quality ratings of included studies, based on ISPOR Task Force for Conjoint Analysis checklist. **Appendix 6.** Number of studies that examined attributes within various levels, dimensions, and features of primary care according to the types of visits. **Appendix 7.** Full list of attributes according to evidence levels, overall and by types of visits (main analyses). **Appendix 8.** Full list of factors affecting preference heterogeneity according to evidence levels, overall and by types of visits (main analyses).

## Data Availability

All data presented in the manuscript or additional files are extracted from published papers, hence are publicly available.

## Abbreviations

DCE: Discrete choice experiment
PRISMA: Preferred Reporting items for systematic reviews and meta-analyses
LMIC: Low and middle-income countries
ISPOR: International society for pharmacoeconomics and outcomes research
PC Monitor: Primary care monitoring system
