# Supplementary material for: Conjoint analyses of patients’ preferences for primary care: a systematic review
Source: BMC Prim Care. 2022 Sep 9;23:234. doi: 10.1186/s12875-022-01822-8 (PMC9463739; doi:10.1186/s12875-022-01822-8)
Supplement: Supplementary file 1 — Additional file 1: Appendix 1. PRISMA checklist. Appendix 2. Search strategies. Appendix 3. List of inclusion and exclusion criteria. Appendix 4. Detailed characteristics of included studies (include quality rating for each paper). Appendix 5. Methodological quality ratings of included studies, based on ISPOR Task Force for Conjoint Analysis checklist. Appendix 6. Number of studies that examined attributes within various levels, dimensions, and features of primary care according to the types of visits. Appendix 7. Full list of attributes according to evidence levels, overall and by types of visits (main analyses). Appendix 8. Full list of factors affecting preference heterogeneity according to evidence levels, overall and by types of visits (main analyses). [file 12875_2022_1822_MOESM1_ESM.docx]

**BMC Primary Care**

**Title: Conjoint Analyses of Patients’ Preferences for Primary Care: A Systematic Review**

Audrey Huili LIM^1^, Sock Wen NG^1^, Xin Rou TEH^1^, Su Miin ONG^1^, Sheamini SIVASAMPU^1^, Ka Keat LIM^2,3^

1. Centre for Clinical Outcomes Research, Institute for Clinical Research, National Institutes of Health, Ministry of Health Malaysia
2. School of Population Health & Environmental Sciences, Faculty of Life Sciences & Medicine, King’s College London, London, United Kingdom.
3. National Institute for Health Research (NIHR) Biomedical Research Centre, Guy's and St Thomas' NHS Foundation Trust and King's College London, United Kingdom

# Appendix 1 PRISMA checklist

| Section and Topic | Item # | Checklist item | Location where item is reported |
| --- | --- | --- | --- |
| TITLE | | |  |
| Title | 1 | Identify the report as a systematic review. | 1 |
| ABSTRACT | | |  |
| Abstract | 2 | See the PRISMA 2020 for Abstracts checklist. | 4 |
| INTRODUCTION | | |  |
| Rationale | 3 | Describe the rationale for the review in the context of existing knowledge. | 7 |
| Objectives | 4 | Provide an explicit statement of the objective(s) or question(s) the review addresses. | 8 |
| METHODS | | |  |
| Eligibility criteria | 5 | Specify the inclusion and exclusion criteria for the review and how studies were grouped for the syntheses. | 9 |
| Information sources | 6 | Specify all databases, registers, websites, organisations, reference lists and other sources searched or consulted to identify studies. Specify the date when each source was last searched or consulted. | 9 |
| Search strategy | 7 | Present the full search strategies for all databases, registers and websites, including any filters and limits used. | Appendix 2 |
| Selection process | 8 | Specify the methods used to decide whether a study met the inclusion criteria of the review, including how many reviewers screened each record and each report retrieved, whether they worked independently, and if applicable, details of automation tools used in the process. | 9 |
| Data collection process | 9 | Specify the methods used to collect data from reports, including how many reviewers collected data from each report, whether they worked independently, any processes for obtaining or confirming data from study investigators, and if applicable, details of automation tools used in the process. | 10 |
| Data items | 10a | List and define all outcomes for which data were sought. Specify whether all results that were compatible with each outcome domain in each study were sought (e.g. for all measures, time points, analyses), and if not, the methods used to decide which results to collect. | 10 |
|  | 10b | List and define all other variables for which data were sought (e.g. participant and intervention characteristics, funding sources). Describe any assumptions made about any missing or unclear information. | 10 |
| Study risk of bias assessment | 11 | Specify the methods used to assess risk of bias in the included studies, including details of the tool(s) used, how many reviewers assessed each study and whether they worked independently, and if applicable, details of automation tools used in the process. | 10 |
| Effect measures | 12 | Specify for each outcome the effect measure(s) (e.g. risk ratio, mean difference) used in the synthesis or presentation of results. | 11 |
| Synthesis methods | 13a | Describe the processes used to decide which studies were eligible for each synthesis (e.g. tabulating the study intervention characteristics and comparing against the planned groups for each synthesis (item #5)). | 11 |
|  | 13b | Describe any methods required to prepare the data for presentation or synthesis, such as handling of missing summary statistics, or data conversions. | 11 |
|  | 13c | Describe any methods used to tabulate or visually display results of individual studies and syntheses. | 11 |
|  | 13d | Describe any methods used to synthesize results and provide a rationale for the choice(s). If meta-analysis was performed, describe the model(s), method(s) to identify the presence and extent of statistical heterogeneity, and software package(s) used. | 11-12 |
|  | 13e | Describe any methods used to explore possible causes of heterogeneity among study results (e.g. subgroup analysis, meta-regression). | 11-12 |
|  | 13f | Describe any sensitivity analyses conducted to assess robustness of the synthesized results. | 11-12 |
| Reporting bias assessment | 14 | Describe any methods used to assess risk of bias due to missing results in a synthesis (arising from reporting biases). | NA |
| Certainty assessment | 15 | Describe any methods used to assess certainty (or confidence) in the body of evidence for an outcome. | NA |
| RESULTS | | |  |
| Study selection | 16a | Describe the results of the search and selection process, from the number of records identified in the search to the number of studies included in the review, ideally using a flow diagram. | 14 |
|  | 16b | Cite studies that might appear to meet the inclusion criteria, but which were excluded, and explain why they were excluded. | 14 |
| Study characteristics | 17 | Cite each included study and present its characteristics. | Appendix 4 |
| Risk of bias in studies | 18 | Present assessments of risk of bias for each included study. | Appendix 5 |
| Results of individual studies | 19 | For all outcomes, present, for each study: (a) summary statistics for each group (where appropriate) and (b) an effect estimate and its precision (e.g. confidence/credible interval), ideally using structured tables or plots. | Appendix 6 & 7 |
| Results of syntheses | 20a | For each synthesis, briefly summarise the characteristics and risk of bias among contributing studies. | 15-16 |
|  | 20b | Present results of all statistical syntheses conducted. If meta-analysis was done, present for each the summary estimate and its precision (e.g. confidence/credible interval) and measures of statistical heterogeneity. If comparing groups, describe the direction of the effect. | 15-16 |
|  | 20c | Present results of all investigations of possible causes of heterogeneity among study results. | 15-16 |
|  | 20d | Present results of all sensitivity analyses conducted to assess the robustness of the synthesized results. | 15-16 |
| Reporting biases | 21 | Present assessments of risk of bias due to missing results (arising from reporting biases) for each synthesis assessed. | NA |
| Certainty of evidence | 22 | Present assessments of certainty (or confidence) in the body of evidence for each outcome assessed. | NA |
| DISCUSSION | | |  |
| Discussion | 23a | Provide a general interpretation of the results in the context of other evidence. | 18 |
|  | 23b | Discuss any limitations of the evidence included in the review. | 20-21 |
|  | 23c | Discuss any limitations of the review processes used. | 20-21 |
|  | 23d | Discuss implications of the results for practice, policy, and future research. | 21-22 |
| OTHER INFORMATION | | |  |
| Registration and protocol | 24a | Provide registration information for the review, including register name and registration number, or state that the review was not registered. | 9 |
|  | 24b | Indicate where the review protocol can be accessed, or state that a protocol was not prepared. | 9 |
|  | 24c | Describe and explain any amendments to information provided at registration or in the protocol. | NA |
| Support | 25 | Describe sources of financial or non-financial support for the review, and the role of the funders or sponsors in the review. | 22 |
| Competing interests | 26 | Declare any competing interests of review authors. | 22 |
| Availability of data, code and other materials | 27 | Report which of the following are publicly available and where they can be found: template data collection forms; data extracted from included studies; data used for all analyses; analytic code; any other materials used in the review. | NA |

*From:*  Page MJ, McKenzie JE, Bossuyt PM, Boutron I, Hoffmann TC, Mulrow CD, et al. The PRISMA 2020 statement: an updated guideline for reporting systematic reviews. BMJ 2021;372:n71. doi: 10.1136/bmj.n71

For more information, visit: <http://www.prisma-statement.org/>

# Appendix 2 Search strategies

## PubMed

| **Search number** | **Query** | **Results** |
| --- | --- | --- |
| 50 | (((((patient preference*[Title/Abstract]) OR (patient preference[MeSH Terms])) OR (patient priorit*[Title/Abstract])) OR (public preference*[Title/Abstract])) OR ((((((((discrete choice[Title/Abstract]) OR (DCE[Title/Abstract])) OR (conjoint[Title/Abstract])) OR (functional measurement*[Title/Abstract])) OR (pairwise choice*[Title/Abstract])) OR (paired comparison*[Title/Abstract])) OR (part worth utilit*[Title/Abstract])) OR (stated preference*[Title/Abstract]))) AND (((((((((((((((((((((((((((((((((ambulatory care[Title/Abstract]) OR (ambulatory care facilit*[Title/Abstract])) OR (ambulatory care[MeSH Terms])) OR (ambulatory care facilities[MeSH Terms])) OR (ambulatory health cent*[Title/Abstract])) OR (ambulatory health facilit*[Title/Abstract])) OR (community clinic*[Title/Abstract])) OR (community health cent*[Title/Abstract])) OR (community health centers[MeSH Terms])) OR (community health clinic*[Title/Abstract])) OR (community health service*[Title/Abstract])) OR (community health services[MeSH Terms])) OR (community medicine[Title/Abstract])) OR (community medicine[MeSH Terms])) OR (family health[Title/Abstract])) OR (family medicine[Title/Abstract])) OR (family physician*[Title/Abstract])) OR (family physician[MeSH Terms])) OR (family pract*[Title/Abstract])) OR (general practice physician[MeSH Terms])) OR (general practice*[Title/Abstract])) OR (general practice[MeSH Terms])) OR (general practitioner*[Title/Abstract])) OR (general practitioner[MeSH Terms])) OR (GP*[Title/Abstract])) OR (health cent*[Title/Abstract])) OR (mobile health units[MeSH Terms])) OR (outpatient[Title/Abstract])) OR (primary care[Title/Abstract])) OR (primary health[Title/Abstract])) OR (primary health care[Title/Abstract])) OR (primary health care[MeSH Terms])) OR (primary medical care[Title/Abstract])) | 5,429 |
| 49 | ((((patient preference*[Title/Abstract]) OR (patient preference[MeSH Terms])) OR (patient priorit*[Title/Abstract])) OR (public preference*[Title/Abstract])) OR ((((((((discrete choice[Title/Abstract]) OR (DCE[Title/Abstract])) OR (conjoint[Title/Abstract])) OR (functional measurement*[Title/Abstract])) OR (pairwise choice*[Title/Abstract])) OR (paired comparison*[Title/Abstract])) OR (part worth utilit*[Title/Abstract])) OR (stated preference*[Title/Abstract])) | 33,325 |
| 48 | ((((((((((((((((((((((((((((((((ambulatory care[Title/Abstract]) OR (ambulatory care facilit*[Title/Abstract])) OR (ambulatory care[MeSH Terms])) OR (ambulatory care facilities[MeSH Terms])) OR (ambulatory health cent*[Title/Abstract])) OR (ambulatory health facilit*[Title/Abstract])) OR (community clinic*[Title/Abstract])) OR (community health cent*[Title/Abstract])) OR (community health centers[MeSH Terms])) OR (community health clinic*[Title/Abstract])) OR (community health service*[Title/Abstract])) OR (community health services[MeSH Terms])) OR (community medicine[Title/Abstract])) OR (community medicine[MeSH Terms])) OR (family health[Title/Abstract])) OR (family medicine[Title/Abstract])) OR (family physician*[Title/Abstract])) OR (family physician[MeSH Terms])) OR (family pract*[Title/Abstract])) OR (general practice physician[MeSH Terms])) OR (general practice*[Title/Abstract])) OR (general practice[MeSH Terms])) OR (general practitioner*[Title/Abstract])) OR (general practitioner[MeSH Terms])) OR (GP*[Title/Abstract])) OR (health cent*[Title/Abstract])) OR (mobile health units[MeSH Terms])) OR (outpatient[Title/Abstract])) OR (primary care[Title/Abstract])) OR (primary health[Title/Abstract])) OR (primary health care[Title/Abstract])) OR (primary health care[MeSH Terms])) OR (primary medical care[Title/Abstract]) | 931,336 |
| 47 | primary medical care[Title/Abstract] | 942 |
| 46 | primary health care[MeSH Terms] | 178,165 |
| 45 | primary health care[Title/Abstract] | 30,501 |
| 44 | primary health[Title/Abstract] | 34,031 |
| 43 | primary care[Title/Abstract] | 129,988 |
| 42 | outpatient[Title/Abstract] | 156,655 |
| 41 | mobile health units[MeSH Terms] | 3,802 |
| 40 | health cent*[Title/Abstract] | 34,236 |
| 39 | GP*[Title/Abstract] | 46,454 |
| 38 | general practitioner[MeSH Terms] | 9,298 |
| 37 | general practitioner*[Title/Abstract] | 53,927 |
| 36 | general practice[MeSH Terms] | 77,017 |
| 35 | general practice*[Title/Abstract] | 44,989 |
| 34 | general practice physician[MeSH Terms] | 9,298 |
| 33 | family pract*[Title/Abstract] | 10,494 |
| 32 | family physician[MeSH Terms] | 16,842 |
| 31 | family physician*[Title/Abstract] | 15,559 |
| 30 | family medicine[Title/Abstract] | 12,251 |
| 29 | family health[Title/Abstract] | 6,872 |
| 28 | community medicine[MeSH Terms] | 2,053 |
| 27 | community medicine[Title/Abstract] | 1,752 |
| 26 | community health services[MeSH Terms] | 320,472 |
| 25 | community health service*[Title/Abstract] | 1,972 |
| 24 | community health clinic*[Title/Abstract] | 403 |
| 23 | community health centers[MeSH Terms] | 12,763 |
| 22 | community health cent*[Title/Abstract] | 4,506 |
| 21 | community clinic*[Title/Abstract] | 2,399 |
| 20 | ambulatory health facilit*[Title/Abstract] | 8 |
| 19 | ambulatory health cent*[Title/Abstract] | 34 |
| 18 | ambulatory care facilities[MeSH Terms] | 57,557 |
| 17 | ambulatory care[MeSH Terms] | 55,271 |
| 16 | ambulatory care facilit*[Title/Abstract] | 250 |
| 15 | ambulatory care[Title/Abstract] | 10,587 |
| 14 | (((((((discrete choice[Title/Abstract]) OR (DCE[Title/Abstract])) OR (conjoint[Title/Abstract])) OR (functional measurement*[Title/Abstract])) OR (pairwise choice*[Title/Abstract])) OR (paired comparison*[Title/Abstract])) OR (part worth utilit*[Title/Abstract])) OR (stated preference*[Title/Abstract]) | 15,770 |
| 13 | stated preference*[Title/Abstract] | 875 |
| 12 | part worth utilit*[Title/Abstract] | 45 |
| 11 | paired comparison*[Title/Abstract] | 2,602 |
| 10 | pairwise choice*[Title/Abstract] | 51 |
| 9 | functional measurement*[Title/Abstract] | 1,072 |
| 8 | conjoint[Title/Abstract] | 3,145 |
| 7 | DCE[Title/Abstract] | 6,864 |
| 6 | discrete choice[Title/Abstract] | 2,617 |
| 5 | (((patient preference*[Title/Abstract]) OR (patient preference[MeSH Terms])) OR (patient priorit*[Title/Abstract])) OR (public preference*[Title/Abstract]) | 18,936 |
| 4 | public preference*[Title/Abstract] | 345 |
| 3 | patient priorit*[Title/Abstract] | 538 |
| 2 | patient preference[MeSH Terms] | 9,983 |
| 1 | patient preference*[Title/Abstract] | 10,500 |

## Scopus

| **#** | **Search terms** | **Results** |
| --- | --- | --- |
| 40 | ( ( TITLE-ABS-KEY ( "ambulatory care" ) )  OR  ( TITLE-ABS-KEY ( "ambulatory care facilit*" ) )  OR  ( TITLE-ABS-KEY ( "ambulatory health cent*" ) )  OR  ( TITLE-ABS-KEY ( "ambulatory health facilit*" ) )  OR  ( TITLE-ABS-KEY ( "community clinic*" ) )  OR  ( TITLE-ABS-KEY ( "community health cent*" ) )  OR  ( TITLE-ABS-KEY ( "community health clinic*" ) )  OR  ( TITLE-ABS-KEY ( "community health service*" ) )  OR  ( TITLE-ABS-KEY ( "community medicine" ) )  OR  ( TITLE-ABS-KEY ( "family doctor*" ) )  OR  ( TITLE-ABS-KEY ( "family health" ) )  OR  ( TITLE-ABS-KEY ( "family medicine" ) )  OR  ( TITLE-ABS-KEY ( "family physician*" ) )  OR  ( TITLE-ABS-KEY ( "family pract*" ) )  OR  ( TITLE-ABS-KEY ( "general practice*" ) )  OR  ( TITLE-ABS-KEY ( "general practitioner*" ) )  OR  ( TITLE-ABS-KEY ( "GP*" ) )  OR  ( TITLE-ABS-KEY ( "general practice physician" ) )  OR  ( TITLE-ABS-KEY ( "health cent*" ) )  OR  ( TITLE-ABS-KEY ( "mobile health units" ) )  OR  ( TITLE-ABS-KEY ( "outpatient" ) )  OR  ( TITLE-ABS-KEY ( "primary care" ) )  OR  ( TITLE-ABS-KEY ( "primary health" ) )  OR  ( TITLE-ABS-KEY ( "primary health care" ) )  OR  ( TITLE-ABS-KEY ( "primary medical care" ) ) )  AND  ( ( ( TITLE-ABS-KEY ( "patient preference*" )  OR  TITLE-ABS-KEY ( "patient priorit*" )  OR  TITLE-ABS-KEY ( "public preference*" ) ) )  OR  ( ( TITLE-ABS-KEY ( "discrete choice" )  OR  TITLE-ABS-KEY ( "DCE" )  OR  TITLE-ABS-KEY ( "conjoint" )  OR  TITLE-ABS-KEY ( "functional measurement*" )  OR  TITLE-ABS-KEY ( "pairwise choice*" )  OR  TITLE-ABS-KEY ( "paired comparison*" )  OR  TITLE-ABS-KEY ( "part worth utilit*" )  OR  TITLE-ABS-KEY ( "stated preference*" ) ) ) ) | 5491 |
| 39 | TITLE-ABS-KEY ( "primary medical care" ) | 82872 |
| 38 | TITLE-ABS-KEY ( "primary health care" ) | 118297 |
| 37 | TITLE-ABS-KEY ( "primary health" ) | 121637 |
| 36 | TITLE-ABS-KEY ( "primary care" ) | 151443 |
| 35 | TITLE-ABS-KEY ( "outpatient" ) | 284200 |
| 34 | TITLE-ABS-KEY ( "mobile health units" ) | 3509 |
| 33 | TITLE-ABS-KEY ( "health cent*" ) | 71817 |
| 32 | TITLE-ABS-KEY ( "general practice physician" ) | 136 |
| 31 | TITLE-ABS-KEY ( "GP*" ) | 580960 |
| 30 | TITLE-ABS-KEY ( "general practitioner*" ) | 125403 |
| 29 | TITLE-ABS-KEY ( "general practice*" ) | 112173 |
| 28 | TITLE-ABS-KEY ( "family pract*" ) | 65805 |
| 27 | TITLE-ABS-KEY ( "family physician*" ) | 19365 |
| 26 | TITLE-ABS-KEY ( "family medicine" ) | 19059 |
| 25 | TITLE-ABS-KEY ( "family health" ) | 30749 |
| 24 | TITLE-ABS-KEY ( "family doctor*" ) | 7336 |
| 23 | TITLE-ABS-KEY ( "community medicine" ) | 5633 |
| 22 | TITLE-ABS-KEY ( "community health service*" ) | 32227 |
| 21 | TITLE-ABS-KEY ( "community health clinic*" ) | 455 |
| 20 | TITLE-ABS-KEY ( "community health cent*" ) | 10940 |
| 19 | TITLE-ABS-KEY ( "community clinic" ) | 1991 |
| 18 | TITLE-ABS-KEY ( "ambulatory health facilit*" ) | 5 |
| 17 | TITLE-ABS-KEY ( "ambulatory health cent*" ) | 42 |
| 16 | TITLE-ABS-KEY ( "ambulatory care facilit*" ) | 17765 |
| 15 | TITLE-ABS-KEY ( "ambulatory care" ) | 78627 |
| 14 | ( TITLE-ABS-KEY ( "patient preference*" )  OR  TITLE-ABS-KEY ( "patient priorit*" )  OR  TITLE-ABS-KEY ( "public preference*" )  OR  TITLE-ABS-KEY ( "discrete choice" )  OR  TITLE-ABS-KEY ( "DCE" )  OR  TITLE-ABS-KEY ( "conjoint" )  OR  TITLE-ABS-KEY ( "functional measurement*" )  OR  TITLE-ABS-KEY ( "pairwise choice*" )  OR  TITLE-ABS-KEY ( "paired comparison*" )  OR  TITLE-ABS-KEY ( "part worth utilit*) OR TITLE-ABS-KEY("  stated  AND preference* ) ) | 68656 |
| 13 | ( TITLE-ABS-KEY ( "discrete choice" ) )  OR  ( TITLE-ABS-KEY ( "DCE" ) )  OR  ( TITLE-ABS-KEY ( "conjoint" ) )  OR  ( TITLE-ABS-KEY ( "functional measurement*" ) )  OR  ( TITLE-ABS-KEY ( "pairwise choice*" ) )  OR  ( TITLE-ABS-KEY ( "paired comparison*" ) )  OR  ( TITLE-ABS-KEY ( "part worth utilit*" ) )  OR  ( TITLE-ABS-KEY ( "stated preference*" ) ) | 41392 |
| 12 | TITLE-ABS-KEY ( "stated preference*" ) | 5188 |
| 11 | TITLE-ABS-KEY ( "part worth utilit*" ) | 132 |
| 10 | TITLE-ABS-KEY ( "paired comparison*" ) | 6314 |
| 9 | TITLE-ABS-KEY ( "pairwise choice*" ) | 167 |
| 8 | TITLE-ABS-KEY ( "functional measurement*" ) | 1488 |
| 7 | TITLE-ABS-KEY ( "conjoint" ) | 10087 |
| 6 | TITLE-ABS-KEY ( "DCE" ) | 11089 |
| 5 | TITLE-ABS-KEY ( "discrete choice" ) | 9976 |
| 4 | ( TITLE-ABS-KEY ( "patient preference*" ) )  OR  ( TITLE-ABS-KEY ( "patient priorit*" ) )  OR  ( TITLE-ABS-KEY ( "public preference*" ) ) | 32750 |
| 3 | TITLE-ABS-KEY ( "public preference*" ) | 1511 |
| 2 | TITLE-ABS-KEY ( "patient priorit*" ) | 1123 |
| 1 | TITLE-ABS-KEY ( "patient preference*" ) | 30,340 |

## Embase

| No. | Query | Results |
| --- | --- | --- |
| #52 | #16 AND #51 | 4448 |
| #51 | #17 OR #18 OR #19 OR #20 OR #21 OR #22 OR #23 OR #24 OR #25 OR #26 OR #27 OR #28 OR #29 OR #30 OR #31 OR #32 OR #33 OR #34 OR #35 OR #36 OR #37 OR #38 OR #39 OR #40 OR #41 OR #42 OR #43 OR #44 OR #45 OR #46 OR #47 OR #48 OR #49 OR #50 | 1079228 |
| #50 | 'outpatient'/exp | 140315 |
| #49 | outpatient:ab,ti | 260395 |
| #48 | 'primary medical care'/exp | 115723 |
| #47 | 'primary medical care':ab,ti | 1167 |
| #46 | 'primary health care'/exp | 186321 |
| #45 | 'primary health care':ab,ti | 27572 |
| #44 | 'primary health':ab,ti | 32171 |
| #43 | 'primary care':ab,ti | 171173 |
| #42 | 'health center'/exp | 37698 |
| #41 | 'health cent*':ab,ti | 44467 |
| #40 | 'general practice physician':ab,ti | 34 |
| #39 | gp*:ab,ti | 291666 |
| #38 | 'general practice'/exp | 87667 |
| #37 | 'general practice*':ab,ti | 51950 |
| #36 | 'general practitioner'/exp | 108115 |
| #35 | 'general practitioner*':ab,ti | 72333 |
| #34 | 'family pract*':ab,ti | 11327 |
| #33 | 'family physician':ab,ti | 6764 |
| #32 | 'family medicine'/exp | 11962 |
| #31 | 'family medicine':ab,ti | 14486 |
| #30 | 'family health'/exp | 10859 |
| #29 | 'family health':ab,ti | 7320 |
| #28 | 'family doctor*' | 8574 |
| #27 | 'community medicine'/exp | 2859 |
| #26 | 'community medicine':ab,ti | 1945 |
| #25 | 'community health service*':ab,ti | 1670 |
| #24 | 'community health clinic*':ab,ti | 516 |
| #23 | 'community health cent*':ab,ti | 5774 |
| #22 | 'community clinic*':ab,ti | 3477 |
| #21 | 'ambulatory health facilit*':ab,ti | 5 |
| #20 | 'ambulatory health cent*':ab,ti | 48 |
| #19 | 'ambulatory care facilit*':ab,ti | 230 |
| #18 | 'ambulatory care'/exp | 53622 |
| #17 | 'ambulatory care':ab,ti | 12994 |
| #16 | #5 OR #15 | 48020 |
| #15 | #6 OR #7 OR #8 OR #9 OR #10 OR #11 OR #12 OR #13 OR #14 | 20018 |
| #14 | 'stated preference*':ab,ti | 981 |
| #13 | 'part worth utilit*':ab,ti | 86 |
| #12 | 'paired comparison':ab,ti | 1928 |
| #11 | 'pairwise choice*':ab,ti | 50 |
| #10 | 'functional measurement*':ab,ti | 1501 |
| #9 | conjoint:ab,ti | 3868 |
| #8 | 'discrete choice experiment'/exp | 239 |
| #7 | dce:ab,ti | 10173 |
| #6 | 'discrete choice':ab,ti | 3618 |
| #5 | #1 OR #2 OR #3 OR #4 | 29722 |
| #4 | 'public preference*':ab,ti | 405 |
| #3 | 'patient priorit*':ab,ti | 787 |
| #2 | 'patient preference'/exp | 22107 |
| #1 | 'patient preference*':ab,ti | 15090 |

## Econlit

| # | Searches | Results |
| --- | --- | --- |
| 44 | 14 and 43 | 373 |
| 43 | 15 or 16 or 17 or 18 or 19 or 20 or 21 or 22 or 23 or 24 or 25 or 26 or 27 or 28 or 29 or 30 or 31 or 32 or 33 or 34 or 35 or 36 or 37 or 38 or 39 or 40 or 41 or 42 | 10520 |
| 42 | [exp Health Care Services/ or exp Primary Health Care/ or exp Health Care Utilization/ or exp Health Care Delivery/ or primary medical care.mp.] | 537 |
| 41 | primary health care.mp. [mp=heading words, abstract, title, country as subject] | 537 |
| 40 | primary health.mp. [mp=heading words, abstract, title, country as subject] | 810 |
| 39 | primary care.mp. [mp=heading words, abstract, title, country as subject] | 1239 |
| 38 | outpatient.mp. [mp=heading words, abstract, title, country as subject] | 888 |
| 37 | health cent*.mp. [mp=heading words, abstract, title, country as subject] | 4315 |
| 36 | general practice physician.mp. [mp=heading words, abstract, title, country as subject] | 7 |
| 35 | GP*.mp. [mp=heading words, abstract, title, country as subject] | 2129 |
| 34 | [exp General Practitioners/] | 21 |
| 33 | general practitioner*.mp. [mp=heading words, abstract, title, country as subject] | 369 |
| 32 | [exp Health Care Services/ or exp Primary Health Care/ or exp Family Physicians/ or exp Family Medicine/] | 9 |
| 31 | family pract*.mp. [mp=heading words, abstract, title, country as subject] | 377 |
| 30 | [family physician.mp. or exp Family Physicians/] | 82 |
| 29 | family medicine.mp. [mp=heading words, abstract, title, country as subject] | 58 |
| 28 | family health.mp. [mp=heading words, abstract, title, country as subject] | 1375 |
| 27 | family doctor*.mp. [mp=heading words, abstract, title, country as subject] | 53 |
| 26 | [exp Primary Health Care/ or exp Public Health/ or exp Physicians/ or exp Health Care Delivery/ or exp Health Care Services/ or exp Community Services/] | 12 |
| 25 | [exp Health Promotion/ or exp Community Mental Health Services/ or exp Communities/ or exp Community Services/ or exp Primary Health Care/ or exp Health Service Needs/ or exp Health Care Delivery/ or exp Community Health/ or exp Health Care Services/] | 1 |
| 24 | community health service*.mp. [mp=heading words, abstract, title, country as subject] | 195 |
| 23 | community health clinic*.mp. [mp=heading words, abstract, title, country as subject] | 26 |
| 22 | [exp Health/ or exp Community Mental Health Centers/ or exp Community Mental Health Services/ or exp Health Care Services/ or exp Primary Health Care/ or exp Community Services/ or exp Community Health/ or exp Health Care Delivery/] | 5 |
| 21 | community health cent*.mp. [mp=heading words, abstract, title, country as subject] | 116 |
| 20 | community clinic*.mp. [mp=heading words, abstract, title, country as subject] | 61 |
| 19 | ambulatory health facilit*.mp. [mp=heading words, abstract, title, country as subject] | 2 |
| 18 | ambulatory health cent*.mp. [mp=heading words, abstract, title, country as subject] | 3 |
| 17 | [exp Health Care Delivery/ or exp Health Care Utilization/ or exp Health Care Costs/ or exp Patients/ or exp Health Care Services/ or ambulatory care facilities.mp.] | 3 |
| 16 | ambulatory care facilit*.mp. [mp=heading words, abstract, title, country as subject] | 3 |
| 15 | [ambulatory care.mp. or exp Outpatient Treatment/] | 172 |
| 14 | 12 or 13 | 18730 |
| 13 | 4 or 5 or 6 or 7 or 8 or 9 or 10 or 11 | 10940 |
| 12 | 1 or 2 or 3 | 8592 |
| 11 | stated preference*.mp. [mp=heading words, abstract, title, country as subject] | 2145 |
| 10 | part worth utilit*.mp. [mp=heading words, abstract, title, country as subject] | 37 |
| 9 | paired comparison*.mp. [mp=heading words, abstract, title, country as subject] | 189 |
| 8 | pairwise choice*.mp. [mp=heading words, abstract, title, country as subject] | 324 |
| 7 | functional measurement*.mp. [mp=heading words, abstract, title, country as subject] | 579 |
| 6 | conjoint.mp. [mp=heading words, abstract, title, country as subject] | 886 |
| 5 | DCE.mp. [mp=heading words, abstract, title, country as subject] | 177 |
| 4 | discrete choice.mp. [mp=heading words, abstract, title, country as subject] | 7260 |
| 3 | public preference*.mp. [mp=heading words, abstract, title, country as subject] | 7960 |
| 2 | patient priorit*.mp. [mp=heading words, abstract, title, country as subject] | 258 |
| 1 | patient preference*.mp. [mp=heading words, abstract, title, country as subject] | 614 |

## PsycInfo

| # | Searches | Results |
| --- | --- | --- |
| 44 | 14 and 43 | 3239 |
| 43 | 15 or 16 or 17 or 18 or 19 or 20 or 21 or 22 or 23 or 24 or 25 or 26 or 27 or 28 or 29 or 30 or 31 or 32 or 33 or 34 or 35 or 36 or 37 or 38 or 39 or 40 or 41 or 42 | 774199 |
| 42 | exp Health Care Services/ or exp Primary Health Care/ or exp Health Care Utilization/ or exp Health Care Delivery/ or primary medical care.mp. | 225560 |
| 41 | primary health care.mp. | 25746 |
| 40 | primary health.mp. | 26154 |
| 39 | primary care.mp. | 34347 |
| 38 | outpatient.mp. | 41194 |
| 37 | health cent*.mp. | 12989 |
| 36 | general practice physician.mp. | 8 |
| 35 | GP*.mp. | 20502 |
| 34 | exp General Practitioners/ | 6117 |
| 33 | general practitioner*.mp. | 12272 |
| 32 | exp Health Care Services/ or exp Primary Health Care/ or exp Family Physicians/ or exp Family Medicine/ | 227291 |
| 31 | family pract*.mp. | 6700 |
| 30 | family physician.mp. or exp Family Physicians/ | 2186 |
| 29 | family medicine.mp. | 2634 |
| 28 | family health.mp. | 7075 |
| 27 | family doctor*.mp. | 795 |
| 26 | exp Primary Health Care/ or exp Public Health/ or exp Physicians/ or exp Health Care Delivery/ or exp Health Care Services/ or exp Community Services/ | 314714 |
| 25 | exp Health Promotion/ or exp Community Mental Health Services/ or exp Communities/ or exp Community Services/ or exp Primary Health Care/ or exp Health Service Needs/ or exp Health Care Delivery/ or exp Community Health/ or exp Health Care Services/ | 326328 |
| 24 | community health service*.mp. | 3760 |
| 23 | community health clinic*.mp. | 172 |
| 22 | exp Health/ or exp Community Mental Health Centers/ or exp Community Mental Health Services/ or exp Health Care Services/ or exp Primary Health Care/ or exp Community Services/ or exp Community Health/ or exp Health Care Delivery/ | 579636 |
| 21 | community health cent*.mp. | 1854 |
| 20 | community clinic*.mp. | 1080 |
| 19 | ambulatory health facilit*.mp. | 0 |
| 18 | ambulatory health cent*.mp. | 1 |
| 17 | exp Health Care Delivery/ or exp Health Care Utilization/or exp Health Care Costs/ or exp Patients/ or exp HealthCare Services/ or ambulatory care facilities.mp. | 310904 |
| 16 | ambulatory care facilit*.mp. | 1931 |
| 15 | ambulatory care.mp. or exp Outpatient Treatment/ | 15731 |
| 14 | 12 or 13 | 11190 |
| 13 | 4 or 5 or 6 or 7 or 8 or 9 or 10 or 11 | 7829 |
| 12 | 1 or 2 or 3 | 3698 |
| 11 | stated preference*.mp. | 637 |
| 10 | part worth utilit*.mp. | 30 |
| 9 | paired comparison*.mp. | 2029 |
| 8 | pairwise choice*.mp. | 61 |
| 7 | functional measurement*.mp. | 269 |
| 6 | conjoint.mp. | 3833 |
| 5 | DCE.mp. | 373 |
| 4 | discrete choice.mp | 1242 |
| 3 | public preference*.mp. | 187 |
| 2 | patient priorit*.mp. | 90 |
| 1 | patient preference*.mp. | 3440 |

# Appendix 3 List of inclusion and exclusion criteria

| **Inclusion** | **Exclusion** |
| --- | --- |
| All studies that used DCEs or conjoint analyses to survey the patients or the general public on preferences for primary care outpatient visits | 1. Preferences on specific treatment 2. Preferences on specific services in a clinic 3. Services in hospital outpatient clinics 4. Out-of-hour services |

# Appendix 4 Detailed characteristics of included studies (include quality rating for each paper)

| **No** | **Study Citation (First Author, Year) with DOI, if available** | **Study Characteristics** | **List of Attributes examined** | **List of factors affecting preference heterogeneity examined** | **High Quality (Main Analysis)** | **High Quality (Sensitivity Analysis)** |
| --- | --- | --- | --- | --- | --- | --- |
| **1** | Krinke, K et al. (2019)  DOI: https://doi.org/10.1186/s12875-019-0967-y | Country: Germany  Actual sample size: 904 (46.7% men)  Sample mean age: 56.6 years  Types of visits examined: Primary care situation | Identified by literature review and qualitative research.   - Availability of home visits - Choice of healthcare provider - Distance to practice - time - Modern diagnostic facilities - Opening hours - number of days | NA | Yes | Yes |
| **2** | Liu, Y et al. (2019) DOI: https://doi.org/10.1016/j.socscimed.2019.112396 | Country: China  Actual sample size: 559 (47.7% men)  Sample mean age: -  Types of visits examined: Self perceived minor or severe diseases | Identified by literature review and qualitative research   - Wait for appointment - Cost - Choice of healthcare provider - Personal connection in the facility - General condition of medical equipment - Drug availability - Distance to practice – time - Facility size | - Family members - Income - Insurance - Experience - Employment status - Health Status | Yes | Yes |
| **3** | Cheraghi-Sohi, S et al. (2008)  DOI: https://doi.org/10.1370/afm.816 | Country: England  Actual sample size: 1193 (46.2% men)  Sample mean age: 52.7years  Types of visits examined: Minor physical, urgent physical, ambiguous physical or psychological problem. | Identified by literature review and qualitative research   - Wait for appointment - Cost - Physician's knowledge of the patient - Choice of appointment time – convenience - Doctor's interpersonal manner - Quality of the physical exam | NA | No | No |
| **4** | Hjelmgren, J and Anell,A (2007)  DOI: https://doi.org/10.1016/j.healthpol.2007.02.006 | Country: Sweden  Actual sample size: 1600 (59% men)  Sample mean age: 48.9 years  Types of visits examined: Primary care situation | Identified by literature review and policy   - Cost - Choice of healthcare worker - Involvement in decision making - Wait for appointment - Primary care work model | - Gender - Urban Citizen - Education - Distance to Health Care Centre - Previous Visit - Present Registration with GP - Mobility - Activities of daily living - Usual Activities - Pain Score - Anxious or Depressed | Yes | No |
| **5** | Kruk, ME (2011) DOI: https://doi.org/10.1111/j.1475-6773.2011.01266.x | Country: Liberia  Actual sample size: 1434 (54% men)  Sample mean age: NA  Types of visits examined: NA | Identified by literature review and qualitative research   - Waiting time - clinic - Courtesy and respect - Drug availability - Cost - Quality of the physical exam - Management | - Age - Gender | Yes | Yes |
| **6** | Turner, D et al. (2007) DOI: https://doi.org/10.1258/135581907781543021 | Country: England  Actual sample size: 666 (39% men)  Sample mean age: 58 years  Types of visits examined: Minor familiar symptoms, new symptoms, routine monitoring | Identified by qualitative research   - Choice of healthcare provider - See provider you know - Waiting time - appointment - See person who has information about your medical history | NA | Yes | No |
| **7** | Hole, AR (2008)  DOI: https://doi.org/10.1016/j.jhealeco.2007.11.006 | Country: England  Actual sample size: 409 (% men NA)  Sample mean age: NA  Types of visits examined: Minor skin problem | Identified by qualitative research   - Waiting time - appointment - Cost - Choice of appointment time – convenience - Doctor's interpersonal manner - Doctor's knowledge of the patient - Quality of the physical exam | NA | No | No |
| **8** | Pedersen, LB et al. (2012)  DOI: https://doi.org/10.1016/j.jval.2012.01.002 | Country: Denmark  Actual sample size: 698 (46.7% men)  Sample mean age: 41.9 years  Types of visits examined: NA | Identified by literature review and qualitative research   - Waiting time – telephone - Opening hours – weekend - Waiting time – appointment - Distance to practice - miles/km - Waiting time – clinic - Length of consultation time - Choice of healthcare provider | NA | Yes | Yes |
| **9** | Oliver, D et al. (2019) DOI: https://www.researchgate.net/deref/http%3A%2F%2Fdx.doi.org%2F10.1136%2Fbmjopen-2018-023578 | Country: Canada  Actual sample size: 430 (31% men)  Sample mean age: NA  Types of visits examined: cold, blood in stools, anxiety issues, routine check-up, family member, sudden pain | Identified by literature review and qualitative research   - Waiting time – telephone - Waiting time – appointment - Waiting time – clinic - Choice of appointment time – convenience - Familiarity - Choice of healthcare provider | NA | Yes | Yes |
| **10** | Tinelli, M et al. (2014)  DOI: https://doi.org/10.1093/eurpub/cku082 | Country: Germany, England, Slovenia  Actual sample size: 603 (36.1% men)  Sample mean age: 52 years  Types of visits examined: Non-urgent problem | Identified by literature review and expert opinions   - Amount of information received during consultation - Waiting time – appointment - Waiting time – clinic - Involvement in decision making - Quality of the physical exam | NA | Yes | Yes |
| **11** | Gerard, K et al. (2008) DOI: https://doi.org/10.1258/jhsrp.2007.007087 | Country: England  Actual sample size: 1052 (35.8% men)  Sample mean age: 52 years  Types of visits examined: Itchy rash, chronic weight loss | Identified by literature review, qualitative research and policies   - Choice of healthcare provider - Choice of appointment time – convenience - Waiting time – appointment - Length of consultation time | - Age - Car ownership - Carer status - Employment status - Gender - Health Status |  |  |
| **12** | Ahmed, A and Fincham, JE (2011) DOI: https://doi.org/10.1111/j.1745-7599.2010.00577.x | Country: United States of America  Actual sample size: 493 (32.6% men)  Sample mean age: 48.86 years  Types of visits examined: Influenza, urinary tract infection | Method to identify attributes not reported   - Choice of healthcare provider - Waiting time – appointment - Cost | NA | No | No |
| **13** | Seghieri, C et al (2014)  DOI: https://dx.doi.org/10.1007%2Fs10198-013-0542-8 | Country: Italy  Actual sample size: 3263 (23.2% men)  Sample mean age: 58 years  Types of visits examined: Non-urgent problem | Identified by literature review, qualitative research and expert opinions   - Waiting time – clinic - Choice of healthcare provider - Modern diagnostic facilities | - Advice was given by GP in current visit - Age - Chronic Disease status - Frequency of GP visits - Gender - Health status - Reason to see GP in current visit | Yes | Yes |
| **14** | Wang, X et al. (2020) DOI: https://dx.doi.org/10.3390%2Fijerph17010117 | Country: China  Actual sample size: 307 (48.2% men)  Sample mean age: 62.94 years  Types of visits examined: Diabetes mellitus | Identified by literature review and qualitative research   - Quality of the physical exam - Attention to personal situation - Multidisciplinary care - Experience of care provider - Amount of information received during consultation - Friendliness and helpfulness of staff - Distance to practice - time - Cost | NA | Yes | Yes |
| **15** | Lagarde, M et al. (2015)  DOI: https://doi.org/10.1016/j.healthpol.2014.10.008 | Country: England  Actual sample size: 1706 (46.8% men)  Sample mean age: 46.7 years  Types of visits examined: NA | Identified by literature review and qualitative research   - Opening hours - weekend - Opening hours - extended - Opening hours – lunchtime - Waiting time - appointment - Whether the practice meets your specific health needs - How well the practice knows the health care services (e.g. hospital, community nurses, etc) in your neighborhood | NA | Yes | Yes |
| **16** | Haas, M (2005)  DOI: https://doi.org/10.1071/PY05006 | Country: Australia  Actual sample size: 128 (46.9% men)  Sample mean age: 47.8 years  Types of visits examined: Upper respiratory tract infection, vaccination, minor injury, check-up | Method to identify attributes not reported   - Courtesy and respect - Doctor recognises your pain/distress - Doctor takes notice of what you say about your health - Doctor reassures you - Doctor is trustworthy - Involvement in decision making - Amount of information received during consultation | NA | No | No |
| **17** | Scott, A and Vick, S (1999)  DOI: https://doi.org/10.1111/1467-9485.00124 | Country: Scotland  Actual sample size: 639 (43% men)  Sample mean age: 46 years  Types of visits examined: Mild low back pain, severe low back pain | Method to identify attributes not reported   - Communication skills of healthcare provider - Amount of information received during consultation - Depth of explanation - Involvement in decision making - Waiting time - appointment | - Age - Decision making at last visit - Education - Gender - Health status - Ever had a second opinion - Severity of symptoms | Yes | Yes |
| **18** | Scott, A and Vick, S (1998)  DOI: https://doi.org/10.1016/S0167-6296(97)00035-0Get rights and content | Country: Scotland  Actual sample size: 101 (27% men)  Sample mean age: 36.4 years  Types of visits examined: Mild low back pain, severe low back pain | Identified by literature review   - Communication skills of healthcare provider - Amount of information received during consultation - Depth of explanation - Involvement in decision making - Length of consultation time | - Age - Decision making at last visit - Education - Gender - Health Status - Reason to see GP - Ever had a second opinion - Severity or symptoms - Time since last visit | Yes | Yes |
| **19** | Longo, MF et al. (2006)  DOI: https://www.ncbi.nlm.nih.gov/pmc/articles/PMC1821413/ | Country: Wales  Actual sample size: 565 (% men NA)  Sample mean age: 59 years  Types of visits examined: NA | Identified by literature review   - Communication skills of healthcare provider - Amount of information received during consultation - Depth of explanation - Involvement in decision making - Length of consultation time | NA | Yes | Yes |
| **20** | Mengoni, A et al. (2013)  DOI: https://doi.org/10.6000/1929-6029.2013.02.01.08 | Country: Italy  Actual sample size: 3225 (25% men)  Sample mean age: NA  Types of visits examined: Non-urgent problem | Identified by literature review and qualitative research   - Waiting time - clinic - Involvement in decision making - Amount of information received during consultation | - Advice was given by GP in current visit - Chronic Disease status - The GP listened To You Carefully - The GP Involved You in The Decision - You Trust in Your GP - Living Alone - The GP Works With Other GP - Prior experience putting off seeking care from GP | Yes | Yes |
| **21** | Fung, CH et al. (2005) DOI: https://dx.doi.org/10.1111%2Fj.1475-6773.2005.00395.x | Country: United States of America  Actual sample size: 304 (51% men)  Sample mean age: NA  Types of visits examined: Primary care physician | Identified by literature review   - Limited sickness or injury care (acute care) - Care for ongoing health conditions (chronic care) - Provision of preventive care - Communication skills of healthcare provider - Courtesy and respect | NA | Yes | No |
| **22** | Markham, FW et al. (1999)  DOI: https://doi.org/10.1177/01632789922034365 | Country: United States of America  Actual sample size: 292 (% men NA)  Sample mean age: NA  Types of visits examined: Family medicine office | Identified by literature review and surveys   - Waiting time – clinic - Choice of healthcare provider - Waiting time – referral - Amount of billing problems - Involvement in decision making | NA | No | No |
| **23** | Pedersen, LB et al. (2011)  DOI: https://doi.org/10.1016/S1755-5345(13)70044-7 | Country: Denmark  Actual sample size: 1435 (49.7% men)  Sample mean age: 41.8 years  Types of visits examined: Primary care situation | Method to identify attributes not reported   - Waiting time – telephone - Opening hours – weekend - Waiting time – appointment - Distance to practice - miles/km - Waiting time – clinic - Length of consultation time - Choice of healthcare provider - Cost | - NA | Yes | Yes |
| **24** | Fernandes, OB et al. (2020) DOI: https://doi.org/10.1371/journal.pone.0235165 | Country: Hungary  Actual sample size: 1000 (45% men)  Sample mean age: 46 years  Types of visits examined: Health problem that is of concern but not urgent | Identified by literature review   - Waiting time – appointment - Waiting time - clinic - Amount of information received during consultation - Depth of explanation - Involvement in decision making - Length of consultation time   Cost | - NA | Yes | Yes |
| **25** | Rubin, G (2006) DOI: https://www.ncbi.nlm.nih.gov/pmc/articles/PMC1920713/ | Country: England  Actual sample size: 1153 (33% men)  Sample mean age: 46 years  Types of visits examined: Routine, non-urgent problem | Identified by literature review and qualitative research   - Waiting time – appointment - Choice of healthcare provider - Choice of appointment time – convenience | - Age - Employment - Gender - Person attending - Reason for attendance | Yes | Yes |
| **26** | Jian, E (2020) DOI: https://doi.org/10.3390/ijerph17113987 | Country: China  Actual sample size: 196 (33% men)  Sample mean age: 67 years  Types of visits examined: Minor condition, Severe condition | Identified by literature review and qualitative research   - Type of service - Care provider - Treatment measures - Cost - Travel time | - Region - Income - Gender - Education - Marital status - Employment status | Yes | Yes |
| **27** | Zhu, J (2019)  DOI: https://doi.org/10.1002/hpm.2841 | Country: China  Actual sample size: 885 (49% men)  Sample mean age: NA  Types of visits examined: Common illness diagnosis, Diabetes | Identified by literature review and qualitative research   - Insurance reimbursement - Waiting time reduction - Prior expert treatment - Medicine and equipment in community health sector - Relationship with GP | - Education - Gender - Non-communicable disease - Age - Income - More visits | Yes | Yes |
| **28** | Norwood, P (2021)  DOI: https://doi.org/10.1093/fampra/cmab010 | Country: Portugal  Actual sample size: 517 (48% men)  Sample mean age: NA  Types of visits examined: NA | Identified by literature review and qualitative research   - Waiting time to get an appointment - The GP practice ie easy to reach, with good public transport and close by parking available - Who the appointment is with - Time spent with the GP - User charge | NA | Yes | Yes |
| **29** | Peng, Y (2020)  DOI: https://doi.org/10.2147/PPA.S265093 | Country: China  Actual sample size: 372 (36% men)  Sample mean age: 71 years  Types of visits examined: Mild chronic disease | Identified by literature review, qualitative research and expert opinion   - Type of service - Seniority of healthcare provider - Treatment options - Cost - Distance to practice (min) | NA | Yes | Yes |
| **30** | Caldow, J (2006)  DOI: https://doi.org/10.1111/j.1369-7625.2006.00422.x | Country: Scotland  Actual sample size: 1343  Sample mean age: NA  Types of visits examined: Slightly chesty, irritating cough for 2 weeks | Identified by literature review and qualitative research   - Who you see - Waiting time till appointment - Length of consultation - Continuity of health professional | NA | Yes | Yes |
| **31** | Gerard, K (2012)  DOI: https://doi.org/10.1016/j.jval.2012.02.006 | Country: England  Actual sample size: 451  Sample mean age: 9999  Types of visits examined: Regular review of high blood pressure | Identified by literature review   - Professional's words and explanations about your medicines - Length of consultation - Attention paid by professional to your views about medicines - Health review covers | NA | Yes | Yes |
| **32** | Tinelli, M (2009)  DOI: http://dx.doi.org/10.1211/ijpp/17.05.0004 | Country: Scotland  Actual sample size: 204  Sample mean age: 9999  Types of visits examined: Condition already diagnosed by their GP for which they needed long-term medication | Identified by literature review   - Time spent travelling to and waiting in the surgery, consulting with the GP - Time spent travelling to and waiting in the pharmacy, consulting with the pharmacist - Chance of receiving the ‘best’ treatment - The amount of money you have to spend to get the drug (£) (clinical advice provided + medicine + travel) | NA | Yes | Yes |
| **33** | Gerard, K (2014)  DOI: https://doi.org/10.1111/hex.12193 | Country: England  Actual sample size: 451  Sample mean age: 9999  Types of visits examined: Infection | Identified by qualitative research   - Accessibility - Length of consultation - Professional’s attention paid to your views on your problem/medicine(s) - Help offered by professional | NA | Yes | Yes |
| **34** | McAteer, A (2015)  DOI: https://dx.doi.org/10.3399%2Fbjgp15X685705 | Country: United Kingdom  Actual sample size: 851  Sample mean age: 9999  Types of visits examined: Diarrhea, dizziness, chest pain | Identified by qualitative research and survey   - Action you take - Waiting time - Time available for consultation / treatment - Convenience/availability - Cost - Chance of a satisfactory outcome | NA | Yes | Yes |
| **35** | Epstein, D (2020)  DOI: https://doi.org/10.1016/j.socscimed.2020.113284 | Country: Australia  Actual sample size: 1435  Sample mean age: 44.6  Types of visits examined: NA | Identified by literature review and qualitative research   - Consult time - Waiting time - Compulsory co-payment - Patient satisfaction - Choice of doctor - Suggested voluntary contribution | - Age - Gender - Income level | Yes | Yes |

# Appendix 5 Methodological quality ratings of included studies, based on ISPOR Task Force for Conjoint Analysis checklist.

| **Items** | **[**[**1**](#_ENREF_1)**]** | **[**[**2**](#_ENREF_2)**]** | **[**[**3**](#_ENREF_3)**]** | **[**[**4**](#_ENREF_4)**]** | **[**[**5**](#_ENREF_5)**]** | **[**[**6**](#_ENREF_6)**]** | **[**[**7**](#_ENREF_7)**]** | **[**[**8**](#_ENREF_8)**]** | **[**[**9**](#_ENREF_9)**]** | **[**[**10**](#_ENREF_10)**]** | **[**[**11**](#_ENREF_11)**]** | **[**[**12**](#_ENREF_12)**]** | **[**[**13**](#_ENREF_13)**]** | **[**[**14**](#_ENREF_14)**]** | **[**[**15**](#_ENREF_15)**]** | **[**[**16**](#_ENREF_16)**]** | **[**[**17**](#_ENREF_17)**]** | **[**[**18**](#_ENREF_18)**]** | **[**[**19**](#_ENREF_19)**]** | **[**[**20**](#_ENREF_20)**]** | **[**[**21**](#_ENREF_21)**]** | **[**[**22**](#_ENREF_22)**]** | [[23](#_ENREF_23)] | **[**[**24**](#_ENREF_24)**]** | **[**[**25**](#_ENREF_25)**]** | **[**[**26**](#_ENREF_26)**]** | **[**[**27**](#_ENREF_27)**]** | **[**[**28**](#_ENREF_28)**]** | **[**[**29**](#_ENREF_29)**]** | [[30](#_ENREF_30)] | **[**[**31**](#_ENREF_31)**]** | **[**[**32**](#_ENREF_32)**]** | **[**[**33**](#_ENREF_33)**]** | **[**[**34**](#_ENREF_34)**]** | **[**[**35**](#_ENREF_35)**]** | **Total (Y/P)** |
| --- | --- | --- | --- | --- | --- | --- | --- | --- | --- | --- | --- | --- | --- | --- | --- | --- | --- | --- | --- | --- | --- | --- | --- | --- | --- | --- | --- | --- | --- | --- | --- | --- | --- | --- | --- | --- |
| A | Y | Y | Y | Y | Y | Y | Y | Y | Y | Y | Y | Y | Y | Y | Y | Y | Y | P | Y | Y | Y | Y | Y | Y | Y | Y | Y | Y | Y | Y | Y | Y | Y | Y | Y | 35 |
| A(i) | Y | Y | Y | Y | Y | Y | Y | Y | Y | Y | Y | Y | Y | Y | Y | Y | Y | Y | Y | Y | Y | Y | Y | Y | Y | Y | Y | Y | Y | Y | Y | Y | Y | Y | Y |  |
| A(ii) | Y | Y | Y | Y | Y | Y | Y | Y | Y | Y | Y | Y | Y | Y | Y | Y | Y | Y | Y | Y | Y | Y | Y | Y | Y | Y | Y | Y | Y | Y | Y | Y | Y | Y | Y |  |
| A(iii) | Y | Y | Y | Y | Y | Y | Y | Y | Y | Y | Y | Y | Y | Y | Y | Y | Y | P | Y | Y | Y | Y | Y | Y | Y | Y | Y | Y | Y | Y | Y | Y | Y | Y | Y |  |
| B | Y | Y | P | P | Y | Y | Y | Y | Y | Y | Y | N | Y | Y | Y | N | Y | Y | Y | Y | P | P | N | Y | Y | Y | Y | P | Y | Y | Y | Y | Y | Y | Y | 32 |
| B(iv) | Y | Y | Y | P | Y | Y | Y | Y | Y | Y | Y | N | Y | Y | Y | N | Y | Y | Y | Y | Y | P | N | Y | P | Y | Y | Y | Y | Y | Y | Y | Y | Y | Y |  |
| B(v) | Y | Y | Y | Y | Y | Y | Y | Y | Y | Y | Y | N | Y | Y | Y | N | Y | Y | Y | Y | Y | P | N | Y | P | Y | Y | Y | Y | Y | Y | Y | Y | Y | Y |  |
| B(vi) | Y | Y | Y | P | Y | Y | Y | Y | Y | Y | Y | P | Y | Y | Y | N | Y | Y | Y | Y | N | P | N | Y | Y | Y | Y | N | Y | Y | Y | Y | Y | Y | Y |  |
| C | P | P | P | P | Y | P | P | Y | Y | Y | P | P | P | P | Y | Y | P | P | P | P | P | P | Y | Y | Y | Y | Y | Y | Y | Y | Y | Y | Y | Y | Y | 35 |
| C(vii) | Y | Y | Y | Y | Y | Y | Y | Y | Y | Y | Y | Y | Y | Y | Y | Y | Y | Y | Y | Y | Y | Y | Y | Y | Y | Y | Y | Y | Y | Y | Y | Y | Y | Y | Y |  |
| C(viii) | Y | Y | Y | Y | Y | Y | Y | Y | Y | Y | Y | Y | Y | Y | Y | Y | Y | Y | Y | Y | Y | Y | Y | Y | Y | Y | Y | Y | Y | Y | Y | Y | Y | Y | Y |  |
| C(ix) | N | Y | N | N | Y | N | N | Y | Y | Y | N | N | N | N | N | Y | N | N | N | N | N | N | Y | Y | N | N | Y | N | N | Y | Y | Y | Y | Y | N |  |
| D | Y | P | P | P | Y | P | Y | Y | P | Y | Y | Y | P | P | Y | N | P | P | Y | P | P | N | P | Y | P | P | Y | Y | Y | P | Y | Y | P | P | Y | 33 |
| D(x) | Y | Y | Y | Y | Y | Y | Y | Y | Y | Y | Y | Y | Y | Y | Y | P | Y | Y | Y | Y | N | N | Y | Y | Y | Y | Y | Y | Y | Y | Y | Y | Y | Y | Y |  |
| D(xi) | Y | Y | Y | N | Y | Y | Y | Y | N | Y | Y | Y | N | N | Y | N | N | N | Y | N | N | N | P | Y | N | N | Y | Y | N | N | Y | Y | P | Y | Y |  |
| D(xii) | Y | Y | N | Y | Y | Y | Y | Y | Y | Y | Y | Y | Y | Y | Y | N | Y | Y | Y | Y | Y | Y | Y | Y | Y | Y | Y | Y | Y | Y | Y | Y | Y | N | Y |  |
| E | Y | P | P | P | P | P | P | P | Y | P | P | P | Y | P | P | P | P | Y | Y | Y | P | P | Y | Y | Y | Y | Y | P | Y | Y | P | Y | P | Y | P | 34 |
| E(xiii) | Y | Y | Y | Y | Y | Y | Y | Y | Y | Y | Y | Y | Y | Y | Y | Y | Y | Y | Y | Y | Y | Y | Y | Y | Y | Y | Y | N | Y | N | N | N | N | N | N |  |
| E(xiv) | Y | Y | P | Y | Y | Y | Y | Y | Y | Y | Y | Y | Y | Y | Y | Y | Y | Y | Y | Y | Y | Y | Y | Y | Y | Y | Y | Y | Y | Y | Y | Y | Y | Y | Y |  |
| E(xv) | Y | N | N | N | N | N | N | N | Y | N | N | N | Y | N | N | N | N | Y | Y | Y | N | N | Y | Y | N | N | N | N | N | Y | N | Y | N | Y | N |  |
| F | Y | Y | N | P | Y | Y | Y | Y | Y | Y | Y | P | Y | Y | Y | P | Y | Y | Y | P | Y | P | Y | Y | Y | Y | P | P | Y | Y | Y | Y | Y | Y | Y | 34 |
| F(xvi) | Y | Y | Y | Y | Y | Y | Y | Y | Y | Y | Y | Y | Y | Y | Y | Y | Y | Y | Y | Y | Y | P | Y | Y | Y | Y | Y | Y | Y | Y | Y | Y | Y | Y | Y |  |
| F(xvii) | Y | Y | Y | Y | Y | Y | Y | Y | Y | Y | Y | Y | Y | Y | Y | Y | Y | Y | Y | P | Y | N | Y | Y | Y | Y | N | N | Y | Y | Y | Y | Y | Y | Y |  |
| F(xviii) | P | Y | N | Y | P | Y | Y | Y | Y | Y | Y | P | Y | Y | Y | N | Y | Y | Y | Y | Y | Y | Y | Y | Y | Y | Y | Y | Y | Y | Y | Y | Y | Y | Y |  |
| G | Y | P | Y | P | Y | P | P | P | Y | Y | P | P | P | Y | Y | Y | P | P | Y | P | Y | P | P | Y | Y | Y | P | Y | Y | P | Y | Y | Y | Y | Y | 35 |
| F(xix) | Y | Y | Y | Y | Y | Y | Y | Y | Y | Y | Y | Y | Y | Y | Y | Y | Y | N | Y | Y | Y | Y | Y | Y | Y | Y | Y | Y | Y | P | Y | Y | Y | Y | Y |  |
| F(xx) | Y | Y | Y | Y | Y | Y | N | Y | Y | Y | Y | P | Y | Y | Y | Y | Y | Y | Y | Y | Y | Y | Y | Y | Y | Y | Y | Y | Y | Y | Y | Y | Y | Y | Y |  |
| F(xxi) | Y | P | Y | N | Y | N | N | N | Y | Y | N | Y | N | Y | Y | Y | N | N | Y | N | Y | N | N | Y | Y | Y | N | Y | Y | Y | Y | Y | Y | Y | Y |  |
| H | Y | P | P | P | Y | P | N | P | Y | Y | Y | P | Y | P | P | P | Y | Y | Y | Y | P | N | P | P | Y | Y | Y | P | Y | Y | Y | Y | Y | P | Y | 33 |
| H(xxii) | Y | Y | Y | Y | Y | Y | N | Y | Y | Y | Y | Y | Y | Y | Y | Y | Y | Y | Y | Y | Y | P | Y | Y | Y | Y | Y | Y | Y | Y | Y | Y | Y | Y | Y |  |
| H(xxiii) | Y | N | Y | N | Y | N | N | N | Y | Y | Y | N | Y | N | N | N | Y | Y | Y | Y | Y | N | N | N | Y | Y | Y | N | Y | Y | Y | Y | Y | N | Y |  |
| H(xxiv) | Y | Y | Y | Y | Y | Y | Y | Y | Y | Y | Y | Y | Y | Y | Y | P | Y | Y | Y | Y | P | N | Y | Y | Y | Y | Y | Y | Y | Y | Y | Y | Y | Y | Y |  |
| I | Y | Y | P | Y | Y | P | P | Y | Y | Y | Y | Y | Y | Y | Y | P | Y | Y | Y | Y | P | P | P | Y | Y | Y | Y | Y | Y | P | Y | Y | Y | Y | Y | 35 |
| I(xxv) | Y | Y | Y | N | Y | N | P | Y | Y | Y | Y | Y | Y | Y | Y | N | Y | Y | Y | Y | Y | N | Y | Y | Y | Y | Y | Y | Y | Y | Y | Y | Y | Y | Y |  |
| I(xxvi) | Y | Y | Y | Y | Y | Y | Y | Y | Y | Y | Y | Y | Y | Y | Y | Y | Y | Y | Y | Y | P | P | Y | Y | Y | Y | Y | Y | Y | Y | Y | Y | Y | Y | Y |  |
| I(xxvii) | Y | Y | Y | Y | Y | Y | N | Y | Y | Y | Y | Y | Y | Y | Y | N | Y | Y | Y | Y | Y | Y | N | Y | Y | Y | Y | Y | Y | N | Y | Y | Y | Y | Y |  |
| J | Y | Y | Y | P | Y | Y | Y | Y | Y | Y | Y | Y | Y | Y | Y | P | Y | Y | Y | Y | Y | P | P | Y | Y | Y | Y | Y | Y | P | Y | Y | Y | Y | Y | 35 |
| J(xxviii) | Y | Y | Y | Y | Y | Y | Y | Y | Y | Y | Y | Y | Y | Y | Y | Y | Y | Y | Y | Y | Y | Y | Y | Y | Y | Y | Y | Y | Y | Y | Y | Y | Y | Y | Y |  |
| J(xxix) | Y | Y | Y | Y | Y | Y | Y | Y | Y | Y | Y | Y | Y | Y | Y | P | Y | Y | Y | Y | Y | Y | P | Y | Y | Y | Y | Y | Y | P | Y | Y | Y | Y | Y |  |
| J(xxx) | Y | Y | Y | Y | Y | Y | Y | Y | Y | Y | Y | Y | Y | Y | Y | Y | Y | Y | Y | Y | Y | P | Y | Y | Y | Y | Y | Y | Y | Y | Y | Y | Y | Y | P |  |
| Total main items rated “Yes” | 9 | 5 | 3 | 4 | 2 | 9 | 4 | 5 | 7 | 9 | 9 | 7 | 4 | 7 | 6 | 8 | 3 | 6 | 6 | 9 | 6 | 4 | 1 | 4 | 9 | 9 | 9 | 9 | 7 | 10 | 10 | 10 | 10 | 10 | 10 |  |
| % main items rated “Yes” | 90 | 50 | 30 | 40 | 20 | 90 | 40 | 50 | 70 | 90 | 90 | 70 | 40 | 70 | 60 | 80 | 30 | 60 | 60 | 90 | 60 | 40 | 10 | 40 | 90 | 90 | 90 | 90 | 70 | 100 | 100 | 100 | 100 | 100 | 100 |  |
| Total main items rated “Partial” | 1 | 5 | 6 | 7 | 8 | 1 | 6 | 4 | 3 | 1 | 1 | 3 | 5 | 3 | 4 | 2 | 6 | 4 | 4 | 1 | 4 | 6 | 8 | 6 | 1 | 1 | 1 | 1 | 3 | 0 | 0 | 0 | 0 | 0 | 0 |  |
| % main tems rated “Partial” | 10 | 50 | 60 | 70 | 80 | 10 | 60 | 40 | 30 | 10 | 10 | 30 | 50 | 30 | 40 | 20 | 60 | 40 | 40 | 10 | 40 | 60 | 80 | 60 | 10 | 10 | 10 | 10 | 30 | 0 | 0 | 0 | 0 | 0 | 0 |  |
| Total main items rated “No” | 0 | 0 | 1 | 0 | 0 | 0 | 0 | 1 | 0 | 0 | 0 | 0 | 1 | 0 | 0 | 0 | 2 | 0 | 0 | 0 | 0 | 0 | 2 | 1 | 0 | 0 | 0 | 0 | 0 | 0 | 0 | 0 | 0 | 0 | 0 |  |
| % main items rated “No” | 0 | 0 | 10 | 0 | 0 | 0 | 0 | 10 | 0 | 0 | 0 | 0 | 10 | 0 | 0 | 0 | 20 | 0 | 0 | 0 | 0 | 0 | 20 | 10 | 0 | 0 | 0 | 0 | 0 | 0 | 0 | 0 | 0 | 0 | 0 |  |

| Y | Rated “Yes” |
| --- | --- |
| P | Rated “Partial” |
| N | Rated “No” |

| **Item** | **Description** |
| --- | --- |
| A | Was a well defined research question stated and is DCE an appropriate method for answering it? |
| A(i) | Were a well-defined research question and a testable hypothesis articulated? |
| A(ii) | Was the study perspective described, and was the study placed in a particular decision-making or policy context? |
| A(iii) | What is the rationale for using conjoined analysis to answer the research question? |
| B | Was the choice of attributes and levels supported by evidence? |
| B(iv) | Was attribute identification supported by evidence (literature reviews, focus groups or other scientific methods)? |
| B(v) | Was attribute selection justified and consistent with theory? |
| B(vi) | Was level selection for each attribute justified by the evidence and consistent with the study perspective and hypothesis? |
| C | Was the construction of tasks appropriate? |
| C(vii) | Was the number of attributes in each conjoint task justified (that is, full or partial profile)? |
| C(viii) | Was the number of profiles in each conjoint task justified? |
| C(ix) | Was (should) an opt-out or a status-quo alternative (be) included? |
| D | Was the choice of experimental design justified and evaluated? |
| D(x) | Was the choice of experimental design justified? Were alternative experimental designs considered? |
| D(xi) | Were the properties of the experimental design evaluated? |
| D(xii) | Was the number of conjoint tasks included in the data collection instrument appropriate? |
| E | Were preferences elicited appropriately, given the research question? |
| E(xiii) | Was there sufficient motivation and explanation of conjoint tasks? |
| E(xiv) | Was an appropriate elicitation format (that is, rating, ranking, or choice) used? Did (should) the elicitation format allow for indifference? |
| E(xv) | In addition to preference elicitation, did the conjoint tasks include other qualifying questions (for example, strength of preference, confidence in response, and other methods)? |
| F | Was the data collection instrument designed appropriately? |
| F(xvi) | Was appropriate respondent information collected (such as sociodemographic, attitudinal, health history or status, and treatment experience)? |
| F(xvii) | Were the attributes and levels defined, and was any contextual information provided? |
| F(xviii) | Was the level of burden of the data-collection instrument appropriate? Were respondents encouraged and motivated? |
| G | Was the data collection plan appropriate? |
| F(xix) | Was the sampling strategy justified (for example, sample size, stratification, and recruitment)? |
| F(xx) | Was the mode of administration justified and appropriate (for example, face-to-face, pen-and-paper, web-based)? |
| F(xxi) | Were ethical considerations addressed (for example, recruitment, information and/or consent, compensation)? |
| H | Were statistical analyses and model estimations appropriate? |
| H(xxii) | Were respondent characteristics examined and tested? |
| H(xxiii) | Was the quality of the responses examined (for example, rationality, validity, reliability)? |
| H(xxiv) | Was model estimation conducted appropriately? Were issues of clustering and subgroups handled appropriately? |
| I | Were the results and conclusions valid? |
| I(xxv) | Did study results reflect testable hypotheses and account for statistical uncertainty? |
| I(xxvi) | Were study conclusions supported by the evidence and compared with existing findings in the literature? |
| I(xxvii) | Were study limitations and generalizability adequately discussed? |
| J | Was the study presentation clear, concise and complete? |
| J(xxviii) | Was study importance and research context adequately motivated? |
| J(xxix) | Were the study data-collection instrument and methods described? |
| J(xxx) | Were the study implications clearly stated and understandable to a wide audience? |

# Appendix 6 Number of studies that examined attributes within various levels, dimensions, and features of primary care according to the types of visits

| **Levels, Dimensions & Features of Primary Care^1^** | | | **Number of Studies by Types of Visits** | | | |
| --- | --- | --- | --- | --- | --- | --- |
| **Levels** | **Dimensions** | **Features** | **Minor Acute Conditions** | **Major Acute Conditions** | **Chronic Conditions** | **Non-specific / Other Conditions^2^** |
| Structure | Governance | - Appropriate technology in primary care^3^ | - | - | - | 1 |
|  |  | - Decentralization of primary care management and service development^4^ | 1 | - | - | - |
|  | Others | - Facility size^5^ | 1 | 1 | - | - |
|  | Workforce | - Profile of primary care workforce^6^ | 8 | 3 | 3 | 9 |
| Process | Access to services | - Accommodation of accessibility^7^ | 5 | 3 | - | 8 |
|  |  | - Affordability of primary care services^8^ | 8 | 3 | 5 | 6 |
|  |  | - Geographic accessibility of primary care services^9^ | 2 | 1 | 3 | 4 |
|  |  | - Utilisation of primary care services^10^ | 8 | 2 | 3 | 6 |
|  | Comprehensiveness | - First contact for common health problems^11^ | - | - | - | 1 |
|  |  | - Medical equipment available^12^ | 3 | 1 | 1 | 1 |
|  |  | - Treatment and follow-up of diagnoses^13^ | 6 | 1 | 6 | 2 |
|  | Continuity | - Informational continuity of care^14^ | 2 | 1 | - | 1 |
|  |  | - Longitudinal continuity of care^15^ | - | - | - | 1 |
|  |  | - Relational continuity of care^16^ | 9 | 2 | 2 | 6 |
|  | Coordination | - Integration of primary care *and public health*^17^ | - | - | - | 1 |
|  |  | - Skill-mix of primary care providers^18^ | 1 | - | 1 | 1 |
| Outcome | Quality | - Quality of diagnosis and treatment in primary care^19^ | 9 | 2 | 2 | 6 |
|  |  | - Responsiveness^20^ | 4 | 1 | 2 | 2 |
|  | Efficiency | - Efficiency in performance of primary care workforce^21^ | 16 | 4 | 2 | 13 |

1. *The levels, dimensions and features of primary care are defined according to Kringos et al 2010; Each level consists of several dimensions; each dimension in turn, has several features.*
2. *This includes six studies that specified other reasons for visits e.g., general consultation, annual check-up, and appointments for other family members. The remaining nine studies did not specify the reason for visits.*

*Below are 3 examples of attributes for each feature of primary care, except where only 1 or 2 attributes are available from our included studies:*

1. *Amount of billing problems faced by patients.*
2. *Management of clinic (either run by the government or a non-governmental organisation).*
3. *Land area of the healthcare facility.*
4. *Respondent’s choice of provider, experience or skill level of the provider.*
5. *Convenience of appointment time, home visits, extent of opening hours.*
6. *Cost (out-of-pocket) for patients, insurance reimbursement.*
7. *Distance to practice (miles/km), distance to practice (minutes).*
8. *Drug availability, length of consultation time.*
9. *Limited provision of acute care.*
10. *General condition of medical equipment, availability of modern diagnostic equipment, availability of technical equipment.*
11. *Provision of preventive care, quality of physical exam, type of consultation e.g., acute, chronic.*
12. *Provider’s knowledge of the patient.*
13. *Care for ongoing health conditions.*
14. *Communication skills of the provider, courtesy and respect for the patient, familiarity with the provider.*
15. *Familiarity of the practice with healthcare services in neighbourhood or local services.*
16. *Multidisciplinary care, primary care work model.*
17. *Amount of information received during consultation, depth of explanation, involvement in decision making.*
18. *Attention to personal situation, consideration of patients’ perspective, trustworthiness of the provider.*
19. *Waiting time for referral, waiting time for appointment, waiting time at clinic*

# Appendix 7 Full list of attributes according to evidence levels, overall and by types of visits (main analyses)

| **No** | **Levels > Dimensions > Features of Primary care** | **Attributes** | **Evidence level^1^** | | | | | **Number of studies** | **Direction of Preference for overall evidence level^2^** |
| --- | --- | --- | --- | --- | --- | --- | --- | --- | --- |
|  |  |  | **Overall** | **Acute: Minor Conditions** | **Acute: Major Conditions** | **Chronic Conditions** | **Non-specific / Other Conditions** |  |  |
| **1** | Structure > Governance > Appropriate technology in primary care | Amount of billing problems faced by patients | Limited Evidence | - | - | - | Limited Evidence | 1 | 0 |
| **2** | Structure > Governance > Decentralization of primary care management and service development | Management of clinic by government (vs by NGO) | Limited Evidence | Limited Evidence | - | - | - | 1 | + |
| **3** | Structure > Others > Facility size | Land area of the healthcare facility | Limited Evidence | Limited Evidence | Limited Evidence | - | - | 1 | 0 |
| **4** | Structure > Workforce > Profile of primary care workforce | Ability to choose the providers they see | Strong Evidence | Strong Evidence | Strong Evidence | Limited Evidence | Strong Evidence | 15 | NA |
| **5** | Structure > Workforce > Profile of primary care workforce | Experience or skill level of provider | Strong Evidence | - | - | Strong Evidence | - | 2 | + |
| **6** | Process > Access > Accommodation of accessibility | Availability of home visits | Limited Evidence | - | - | - | Limited Evidence | 1 | + |
| **7** | Process > Access > Accommodation of accessibility | Convenience of appointment time | Strong Evidence | Strong Evidence | Limited Evidence | - | Strong Evidence | 5 | + |
| **8** | Process > Access > Accommodation of accessibility | Opening hours – lunchtime | Limited Evidence | - | - | - | Limited Evidence | 1 | + |
| **9** | Process > Access > Accommodation of accessibility | Opening hours - number of days | Limited Evidence | - | - | - | Limited Evidence | 1 | + |
| **10** | Process > Access > Accommodation of accessibility | Opening hours – weekend | Moderate Evidence | - | - | - | Moderate Evidence | 3 | + |
| **11** | Process > Access > Accommodation of accessibility | Opening hours - extended | Strong Evidence | Limited Evidence | Limited Evidence | - | Strong Evidence | 2 | + |
| **12** | Process > Access > Affordability of primary care services | Cost (out-of-pocket) for patients | Strong Evidence | Strong Evidence | Strong Evidence | Strong Evidence | Strong Evidence | 15 | - |
| **13** | Process > Access > Affordability of primary care services | Voluntary contribution (in addition to out-of-pocket cost) | Limited Evidence | - | - | - | Limited Evidence | 1 | - |
| **14** | Process > Access > Affordability of primary care services | Insurance reimbursement | Limited Evidence | Limited Evidence | - | Limited Evidence | - | 1 | + |
| **15** | Process > Access > Geographic accessibility of primary care services | Distance to practice - miles/km | Moderate Evidence | - | - | - | Moderate Evidence | 3 | - |
| **16** | Process > Access > Geographic accessibility of primary care services | Distance to practice - minutes | Strong Evidence | Strong Evidence | Limited Evidence | Strong Evidence | Limited Evidence | 5 | - |
| **17** | Process > Access > Utilisation of primary care services | Drug availability | Strong Evidence | Strong Evidence | Limited Evidence | - | - | 2 | + |
| **18** | Process > Access > Utilisation of primary care services | Length of consultation time | Strong Evidence | Strong Evidence | Limited Evidence | Limited Evidence | Strong Evidence | 12 | + |
| **19** | Process > Access > Utilisation of primary care services | Treatment approach (i.e., modern, traditional, integrated) | Strong Evidence | Limited Evidence | - | Strong Evidence | - | 2 | NA |
| **20** | Process > Comprehensiveness > First contact for common health problems | Limited provision of acute care | Limited Evidence | - | - | - | Limited Evidence | 1 | 0 |
| **21** | Process > Comprehensiveness > Medical equipment available | General condition of medical equipment | Limited Evidence | Limited Evidence | Limited Evidence | - | - | 1 | + |
| **22** | Process > Comprehensiveness > Medical equipment available | Availability of modern diagnostic equipment | Limited Evidence | Inconclusive Evidence | - | - | Limited Evidence | 2 | + |
| **23** | Process > Comprehensiveness > Medical equipment available | Availability of technical equipment | Limited Evidence | Limited Evidence | - | Limited Evidence | - | 1 | + |
| **24** | Process > Comprehensiveness > Treatment and follow-up of diagnoses | Provision of preventive care | Limited Evidence | - | - | - | Limited Evidence | 1 | 0 |
| **25** | Process > Comprehensiveness > Treatment and follow-up of diagnoses | Quality of the physical exam | Strong Evidence | Strong Evidence | Inconclusive Evidence | Strong Evidence | Inconclusive Evidence | 7 | + |
| **26** | Process > Comprehensiveness > Treatment and follow-up of diagnoses | Type of consultation e.g. acute, chronic | Strong Evidence | Limited Evidence | - | Strong Evidence | - | 2 | NA |
| **27** | Process > Comprehensiveness > Treatment and follow-up of diagnoses | Prior expert treatment | Limited Evidence | Limited Evidence | - | Limited Evidence | - | 1 | + |
| **28** | Process > Continuity > Informational continuity of care | Provider's knowledge of the patient | Limited Evidence | Limited Evidence | Inconclusive Evidence | - | Inconclusive Evidence | 2 | + |
| **29** | Process > Continuity > Longitudinal continuity of care | Care for ongoing health conditions (chronic care) | Limited Evidence | - | - | - | Limited Evidence | 1 | 0 |
| **30** | Process > Continuity > Relational continuity of care | Communication skills of provider | Strong Evidence | Limited Evidence | - | - | Strong Evidence | 4 | + |
| **31** | Process > Continuity > Relational continuity of care | Courtesy and respect for the patient | Strong Evidence | Strong Evidence | - | - | Limited Evidence | 3 | + |
| **32** | Process > Continuity > Relational continuity of care | Familiarity with the provider | Limited Evidence | Limited Evidence | Limited Evidence | - | Limited Evidence | 1 | + |
| **33** | Process > Continuity > Relational continuity of care | Friendliness and helpfulness of staff | Limited Evidence | - | - | Limited Evidence | - | 1 | + |
| **34** | Process > Continuity > Relational continuity of care | Personal connection in the facility | Limited Evidence | Limited Evidence | Limited Evidence | - | - | 1 | 0 |
| **35** | Process > Continuity > Relational continuity of care | See person who has information about your medical history | Limited Evidence | Limited Evidence | - | - | Limited Evidence | 1 | + |
| **36** | Process > Continuity > Relational continuity of care | See provider you know | Strong Evidence | Strong Evidence | - | Limited Evidence | Strong Evidence | 5 | + |
| **37** | Process > Coordination > Integration of primary care – secondary care | Practice knows your local services | Limited Evidence | - | - | - | Limited Evidence | 1 | - |
| **38** | Process > Coordination > Skill mix of primary care providers | Multidisciplinary care | Limited Evidence | - | - | Limited Evidence | - | 1 | + |
| **39** | Process > Coordination > Skill mix of primary care providers | Primary care work model | Limited Evidence | Inconclusive Evidence | - | - | Limited Evidence | 2 | + |
| **40** | Outcome > Quality > Quality of diagnosis and treatment in primary care | Amount of information received during consultation | Strong Evidence | Strong Evidence | - | Limited Evidence | Limited Evidence | 8 | + |
| **41** | Outcome > Quality > Quality of diagnosis and treatment in primary care | Depth of Explanation | Strong Evidence | Strong Evidence | - | Limited Evidence | Limited Evidence | 6 | + |
| **42** | Outcome > Quality > Quality of diagnosis and treatment in primary care | Involvement in decision making | Strong Evidence | Strong Evidence | Inconclusive Evidence | - | Strong Evidence | 10 | + |
| **43** | Outcome > Quality > Quality of diagnosis and treatment in primary care | Likelihood of having illness cured | Strong Evidence | Strong Evidence | Limited Evidence | - | Limited Evidence | 2 | + |
| **44** | Outcome > Quality > Quality of diagnosis and treatment in primary care | Patient satisfaction | Limited Evidence | - | - | - | Limited Evidence | 1 | + |
| **45** | Outcome > Quality > Responsiveness | Attention to personal situation | Limited Evidence | - | - | Limited Evidence | - | 1 | + |
| **46** | Outcome > Quality > Responsiveness | Consideration of patient’s perspective | Strong Evidence | Limited Evidence | Inconclusive Evidence | Limited Evidence | Inconclusive Evidence | 3 | + |
| **47** | Outcome > Quality > Responsiveness | Trustworthiness of the provider | Limited Evidence | Limited Evidence | - | - | - | 1 | + |
| **48** | Outcome > Quality > Responsiveness | Reassurance from the provider | Limited Evidence | Limited Evidence | - | - | - | 1 | + |
| **49** | Outcome > Quality > Responsiveness | Support for emotional distress | Limited Evidence | Limited Evidence | - | - | - | 1 | + |
| **50** | Outcome > Quality > Responsiveness | Provider notices what you say about your health (legitimation) | Limited Evidence | Limited Evidence | - | - | - | 1 | + |
| **51** | Outcome > Quality > Responsiveness | Provider's interpersonal manner | Limited Evidence | Limited Evidence | Inconclusive Evidence | - | Inconclusive Evidence | 2 | + |
| **52** | Outcome > Quality > Responsiveness | Whether the practice meets your specific health needs | Limited Evidence | - | - | - | Limited Evidence | 1 | + |
| **53** | Outcome > Efficiency > Efficiency in performance of primary care workforce | Entire time spent to seek and obtain treatment | Limited Evidence | - | - | Limited Evidence | - | 1 | 0 |
| **54** | Outcome > Efficiency > Efficiency in performance of primary care workforce | Waiting time for referral | Limited Evidence | - | - | - | Limited Evidence | 1 | - |
| **55** | Outcome > Efficiency > Efficiency in performance of primary care workforce | Waiting time for appointment | Strong Evidence | Strong Evidence | Strong Evidence | Limited Evidence | Strong Evidence | 20 | - |
| **56** | Outcome > Efficiency > Efficiency in performance of primary care workforce | Waiting time on telephone | Moderate Evidence | Limited Evidence | Limited Evidence | - | Moderate Evidence | 3 | - |
| **57** | Outcome > Efficiency > Efficiency in performance of primary care workforce | Waiting time at clinic | Strong Evidence | Strong Evidence | Limited Evidence | - | Strong Evidence | 10 | - |
| **58** | Outcome > Efficiency > Efficiency in performance of primary care workforce | Waiting time in general | Limited Evidence | - | - | - | Limited Evidence | 1 | - |

1. *“-” is used when a factor has not been examined in any for the type of visits, hence we were unable to perform any synthesis based on the algorithm presented in Appendix 3.*
2. *Direction of preference for overall evidence level: “0” indicates no association, “+” indicates positive association, “-” indicates negative association.*

# Appendix 8 Full list of factors affecting preference heterogeneity according to evidence levels, overall and by types of visits (main analyses)

| **No** | **Type of Factors** | **Factors affecting heterogeneity** | **Evidence level^1^** | | | | | **Number of Studies** |
| --- | --- | --- | --- | --- | --- | --- | --- | --- |
|  |  |  | **Overall** | **Acute: Minor Conditions** | **Acute: Major Conditions** | **Chronic Conditions** | **Non-specific / Other Conditions** |  |
| **1** | Enabling | Activity if not visiting doctor: Attending College (vs others) | Limited Evidence^2^ | Limited Evidence^2^ | - | - | - | 1 |
| **2** | Enabling | Activity if not visiting doctor: Cleaning house (vs others) | Limited Evidence^2^ | Limited Evidence^2^ | - | - | - | 1 |
| **3** | Enabling | Activity if not visiting doctor: Looking after children (vs others) | Limited Evidence^2^ | Limited Evidence^2^ | - | - | - | 1 |
| **4** | Enabling | Activity if not visiting doctor: Other activity (vs looking after children) | Limited Evidence^2^ | Limited Evidence^2^ | - | - | - | 1 |
| **5** | Enabling | Activity if not visiting doctor: Work (vs others) | Limited Evidence^2^ | Limited Evidence^2^ | - | - | - | 1 |
| **6** | Enabling | Advice was given by GP in current visit: Yes (vs No) | Limited Evidence | Limited Evidence | - | - | - | 2 |
| **7** | Enabling | Car ownership: Yes (vs No) | Limited Evidence | Limited Evidence | - | - | - | 1 |
| **8** | Enabling | Carer status: Yes (vs No) | Limited Evidence^2^ | Limited Evidence^2^ | - | - | - | 1 |
| **9** | Enabling | Current GP works with another GP (vs No) | Limited Evidence | Limited Evidence | - | - | - | 1 |
| **10** | Enabling | Current waiting time at present appointment | Inconclusive Evidence^2^ | Inconclusive Evidence^2^ | - | - | - | 1 |
| **11** | Enabling | Decision making at last visit | Inconclusive evidence | Inconclusive evidence | - | - | - | 1 |
| **12** | Enabling | Distance to Health Care Centre | Limited Evidence | Limited Evidence | - | - | - | 1 |
| **13** | Enabling | Employment status: Employed (vs not working / retired) | Strong Evidence | Limited Evidence | Limited Evidence | Limited Evidence | Conflicting Evidence | 6 |
| **14** | Enabling | Ever had second opinion | Limited Evidence | Limited Evidence | - | - | - | 2 |
| **15** | Enabling | GP Involved You in The Decision: Yes (vs No) | Limited Evidence^2^ | Limited Evidence^2^ | - | - | - | 1 |
| **16** | Enabling | GP Listened to You Carefully: Yes (vs No) | Limited Evidence^2^ | Limited Evidence^2^ | - | - | - | 1 |
| **17** | Enabling | GP provided a lot of information at last visit (vs a little) | Inconclusive Evidence | Inconclusive Evidence | - | - | - | 1 |
| **18** | Enabling | Income level: Medium (vs Low) | Strong Evidence | Strong Evidence | - | Limited Evidence | Limited Evidence | 5 |
| **19** | Enabling | Insurance Type: High Premium (vs no insurance) | Limited Evidence | Limited Evidence | - | - | - | 1 |
| **20** | Enabling | Living Alone: Yes (vs No) | Limited Evidence | Limited Evidence | - | - | - | 1 |
| **21** | Enabling | Present registration with GP: (vs not registered) | Limited Evidence^2^ | - | - | - | Limited Evidence^2^ | 1 |
| **22** | Enabling | Prior experience putting off seeking care from GP: Yes (vs No) | Limited Evidence | Limited Evidence | - | - | - | 2 |
| **23** | Health Behavior | Time since last visit | Inconclusive evidence | Inconclusive evidence | - | - | - | 1 |
| **24** | Health Behavior | Facility visiting experience: higher levels (vs primary level only) | Limited Evidence | Limited Evidence | - | - | - | 1 |
| **25** | Need | Appointment for a child | Limited Evidence | Limited Evidence | - | - | - | 1 |
| **26** | Need | Appointment for another person | Limited Evidence | Limited Evidence | - | - | - | 1 |
| **27** | Need | Chronic disease status: Yes (vs No) | Conflicting Evidence | Conflicting Evidence | - | - | - | 3 |
| **28** | Need | Feeling anxious or depressed (vs not) | Limited Evidence | - | - | - | Limited Evidence | 1 |
| **29** | Need | Frequency of GP Visits in the last year: <3 times (vs > 3 times) | Limited Evidence | Limited Evidence | - | - | - | 2 |
| **30** | Need | Health Status: Poor (vs Good) | Conflicting Evidence | Limited Evidence | Limited Evidence | - | - | 5 |
| **31** | Need | Reason for appointment: Emergency (vs none) | Limited Evidence | - | - | - | Limited Evidence | 1 |
| **32** | Need | Reason for appointment: Long standing physical problem (vs none) | Limited Evidence | - | - | - | Limited Evidence | 1 |
| **33** | Need | Reason for appointment: New problem (vs none) | Limited Evidence | - | - | - | Limited Evidence | 1 |
| **34** | Need | Reason for appointment: Psychological problem (vs none) | Limited Evidence | - | - | - | Limited Evidence | 1 |
| **35** | Need | Reason to see GP in current visit: general / minor illness (vs other) | Inconclusive evidence | Inconclusive evidence | - | - | - | 2 |
| **36** | Need | Severity of symptoms | Limited Evidence | Limited Evidence | - | - | - | 2 |
| **37** | Predisposing | Age | Strong Evidence | Strong Evidence | Limited Evidence | Limited Evidence | Strong Evidence | 10 |
| **38** | Predisposing | Education level | Conflicting Evidence | Conflicting Evidence | - | Limited Evidence | - | 4 |
| **39** | Predisposing | Gender | Strong Evidence | Conflicting Evidence | - | Limited Evidence | Strong Evidence | 7 |
| **40** | Predisposing | Marital Status | Limited Evidence | - | - | Limited Evidence | - | 2 |
| **41** | Predisposing | Number of Family Members | Limited Evidence | Limited Evidence | Limited Evidence | - | - | 1 |
| **42** | Predisposing | Region | Limited Evidence | Limited Evidence | - | Limited Evidence | - | 1 |
| **43** | Predisposing | You Trust in Your GP: Yes (vs No) | Limited Evidence | Limited Evidence | - | - | - | 1 |

1. *“-” is used when a factor has not been examined in any for the type of visits, hence we were unable to perform any synthesis based on the algorithm presented in Appendix 3.*
2. *These are factors with limited or inconclusive evidence of* ***no association****, based on the algorithm presented in Appendix 3*

*.*

# List of the 35 included studies

Of the following 35 studies, 16 were also included in Kleij et al 2017 – (3), (4), (6), (7), (8), (10), (11), (12), (13), (15), (20), (25), (30), (31), (32), (33).

1. Krinke K-S, Tangermann U, Amelung VE, Krauth C. Public preferences for primary care provision in Germany – a discrete choice experiment. BMC Family Practice. 2019;20(1):80.

2. Liu Y, Kong Q, de Bekker-Grob EW. Public preferences for health care facilities in rural China: A discrete choice experiment. Social Science and Medicine. 2019;237:112396.

3. Cheraghi-Sohi S, Hole AR, Mead N, McDonald R, Whalley D, Bower P, et al. What patients want from primary care consultations: a discrete choice experiment to identify patients' priorities. Annals of Family Medicine. 2008;6(2):107-15.

4. Hjelmgren J, Anell A. Population preferences and choice of primary care models: a discrete choice experiment in Sweden. Health Policy. 2007;83(2-3):314-22.

5. Kruk ME, Rockers PC, Tornorlah Varpilah S, Macauley R. Population preferences for health care in Liberia: insights for rebuilding a health system. Health Services Research. 2011;46(6pt2):2057-78.

6. Turner D, Tarrant C, Windridge K, Bryan S, Boulton M, Freeman G, et al. Do patients value continuity of care in general practice? An investigation using stated preference discrete choice experiments. Journal of Health Services Research and Policy. 2007;12(3):132-7.

7. Hole AR. Modelling heterogeneity in patients’ preferences for the attributes of a general practitioner appointment. Journal of Health Economics. 2008;27(4):1078-94.

8. Pedersen LB, Kjær T, Kragstrup J, Gyrd-Hansen D. Do General Practitioners Know Patients' Preferences? An Empirical Study on the Agency Relationship at an Aggregate Level Using a Discrete Choice Experiment. Value in Health. 2012;15(3):514-23.

9. Oliver D, Deal K, Howard M, Qian H, Agarwal G, Guenter D. Patient trade-offs between continuity and access in primary care interprofessional teaching clinics in Canada: a cross-sectional survey using discrete choice experiment. BMJ Open. 2019;9(3):e023578.

10. Tinelli M, Nikoloski Z, Kumpunen S, Knai C, Pribakovic Brinovec R, Warren E, et al. Decision-making criteria among European patients: exploring patient preferences for primary care services. European Journal of Public Health. 2014;25(1):3-9.

11. Gerard K, Salisbury C, Street D, Pope C, Baxter H. Is fast access to general practice all that should matter? A discrete choice experiment of patients' preferences. Journal of Health Services Research and Policy. 2008;13 Suppl 2:3-10.

12. Ahmed A, Fincham JE. Patients' view of retail clinics as a source of primary care: boon for nurse practitioners? Journal of the American Academy of Nurse Practitioners. 2011;23(4):193-9.

13. Seghieri C, Mengoni A, Nuti S. Applying discrete choice modelling in a priority setting: an investigation of public preferences for primary care models. European Journal of Health Economics: Health Economics in Prevention and Care. 2014;15(7):773-85.

14. Wang X, Song K, Zhu P, Valentijn P, Huang Y, Birch S. How Do Type 2 Diabetes Patients Value Urban Integrated Primary Care in China? Results of a Discrete Choice Experiment. International Journal of Environmental Research and Public Health. 2019;17(1):117.

15. Lagarde M, Erens B, Mays N. Determinants of the choice of GP practice registration in England: evidence from a discrete choice experiment. Health Policy. 2015;119(4):427-36.

16. Haas M. The impact of non-health attributes of care on patients' choice of GP. Australian Journal of Primary Health. 2005;11:40-6.

17. Scott A, Vick S. Patients, Doctors and Contracts: An Application of Principal-Agent Theory to the Doctor-Patient Relationship. Scottish Journal of Political Economy. 1999;46(2):111-34.

18. Vick S, Scott A. Agency in health care. Examining patients' preferences for attributes of the doctor-patient relationship. Journal of Health Economics. 1998;17(5):587-605.

19. Longo MF, Cohen DR, Hood K, Edwards A, Robling M, Elwyn G, et al. Involving patients in primary care consultations: assessing preferences using discrete choice experiments. British Journal of General Practice. 2006;56(522):35-42.

20. Mengoni A, Seghieri C, Nuti S. Heterogeneity in Preferences for Primary Care Consultations: Results from a Discrete Choice Experiment. International Journal of Statistics in Medical Research. 2013;2:67-75.

21. Fung CH, Elliott MN, Hays RD, Kahn KL, Kanouse DE, McGlynn EA, et al. Patients' Preferences for Technical versus Interpersonal Quality When Selecting a Primary Care Physician. Health Services Research. 2005;40(4):957-77.

22. Markham FW, Diamond JJ, Hermansen CL. The use of conjoint analysis to study patient satisfaction. Evaluation and the Health Professions. 1999;22(3):371-8.

23. Pedersen LB, Kjær T, Kragstrup J, Gyrd-Hansen D. Does the Inclusion of a Cost Attribute in Forced and Unforced Choices Matter?: Results from a Web Survey Applying the Discrete Choice Experiment. Journal of Choice Modelling. 2011;4(3):88-109.

24. Brito Fernandes Ó, Péntek M, Kringos D, Klazinga N, Gulácsi L, Baji P. Eliciting preferences for outpatient care experiences in Hungary: A discrete choice experiment with a national representative sample. PloS One. 2020;15(7):e0235165.

25. Rubin G, Bate A, George A, Shackley P, Hall N. Preferences for access to the GP: a discrete choice experiment. British Journal of General Practice. 2006;56(531):743-8.

26. Jia E, Gu Y, Peng Y, Li X, Shen X, Jiang M, et al. Preferences of Patients with Non-Communicable Diseases for Primary Healthcare Facilities: A Discrete Choice Experiment in Wuhan, China. International Journal of Environmental Research and Public Health. 2020;17(11):3987.

27. Zhu J, Li J, Zhang Z, Li H. Patients' choice and preference for common disease diagnosis and diabetes care: A discrete choice experiment. The International Journal of Health Planning and Management. 2019;34(4):e1544-e55.

28. Norwood P, Correia I, Heidenreich S, Veiga P, Watson V. Is relational continuity of care as important to people as policy makers think? Preferences for continuity of care in primary care. Family Practice. 2021.

29. Peng Y, Jiang M, Shen X, Li X, Jia E, Xiong J. Preferences for Primary Healthcare Services Among Older Adults with Chronic Disease: A Discrete Choice Experiment. Patient Preference and Adherence. 2020;14:1625-37.

30. Caldow J, Bond C, Ryan M, Campbell NC, Miguel FS, Kiger A, et al. Treatment of minor illness in primary care: a national survey of patient satisfaction, attitudes and preferences regarding a wider nursing role. Health Expectations. 2007;10(1):30-45.

31. Gerard K, Tinelli M, Latter S, Blenkinsopp A, Smith A. Valuing the Extended Role of Prescribing Pharmacist in General Practice: Results from a Discrete Choice Experiment. Value in Health. 2012;15(5):699-707.

32. Tinelli M, Ryan M, Bond C. Patients' preferences for an increased pharmacist role in the management of drug therapy. International Journal of Pharmacy Practice. 2010;17(5):275-82.

33. Gerard K, Tinelli M, Latter S, Smith A, Blenkinsopp A. Patients' valuation of the prescribing nurse in primary care: a discrete choice experiment. Health Expectations. 2015;18(6):2223-35.

34. McAteer A, Yi D, Watson V, Norwood P, Ryan M, Hannaford PC, et al. Exploring preferences for symptom management in primary care: a discrete choice experiment using a questionnaire survey. British Journal of General Practice. 2015;65(636):e478-e88.

35. Epstein DS, Barton C, Mazza D, Woode ME, Mortimer D. Patient chosen gap payments in primary care: Predictions of patient acceptability, uptake and willingness to pay from a discrete choice experiment. Social Science and Medicine. 2020;263:113284.
